# Naturally-Occurring Alkaloids of Plant Origin as Potential Antimicrobials against Antibiotic-Resistant Infections

**DOI:** 10.3390/molecules25163619

**Published:** 2020-08-09

**Authors:** Bruno Casciaro, Laura Mangiardi, Floriana Cappiello, Isabella Romeo, Maria Rosa Loffredo, Antonia Iazzetti, Andrea Calcaterra, Antonella Goggiamani, Francesca Ghirga, Maria Luisa Mangoni, Bruno Botta, Deborah Quaglio

**Affiliations:** 1 Center For Life Nano Science@Sapienza, Istituto Italiano di Tecnologia, Viale Regina Elena 291, 00161 Rome, Italy; bruno.casciaro@iit.it (B.C.); laura.mangiardi@uniroma1.it (L.M.); Isabella.romeo@uniroma1.it (I.R.); 2 Department of Chemistry and Technology of Drugs, “Department of Excellence 2018−2022”, Sapienza University of Rome, P.le Aldo Moro 5, 00185 Rome, Italy; antonia.iazzetti@uniroma1.it (A.I.); andrea.calcaterra@uniroma1.it (A.C.); antonella.goggiamani@uniroma1.it (A.G.); deborah.quaglio@uniroma1.it (D.Q.); 3 Laboratory affiliated to Pasteur Italia-Fondazione Cenci Bolognetti, Department of Biochemical Sciences, Sapienza University of Rome, P.le Aldo Moro 5, 00185 Rome, Italy; floriana.cappiello@uniroma1.it (F.C.); mariarosa.loffredo@uniroma1.it (M.R.L.)

**Keywords:** antibiotic resistance, antimicrobials, alkaloids, methicillin-resistant *Staphylococcus aureus*, vancomycin-resistant enterococci, natural products, plant-derived alkaloids, structure–activity relationship

## Abstract

Antibiotic resistance is now considered a worldwide problem that puts public health at risk. The onset of bacterial strains resistant to conventional antibiotics and the scarcity of new drugs have prompted scientific research to re-evaluate natural products as molecules with high biological and chemical potential. A class of natural compounds of significant importance is represented by alkaloids derived from higher plants. In this review, we have collected data obtained from various research groups on the antimicrobial activities of these alkaloids against conventional antibiotic-resistant strains. In addition, the structure–function relationship was described and commented on, highlighting the high potential of alkaloids as antimicrobials.

## 1. Introduction

The discovery and the advent of penicillin in clinical practice have led to the subsequent discovery of numerous new antibiotics to be used as an invaluable weapon against bacterial infections. However, the beginning of the antibiotic era coincided with the onset and characterization of antibiotic-resistant strains. This triggered the entrance into our current post-antibiotic era in which fewer and fewer antibiotics are discovered at the expense of a high occurrence of multidrug resistant (MDR) infections [1]. Currently in Europe, the number of MDR infections accounts to 700 thousand and provokes 33,000 deaths every year, resulting in an estimated cost of above €1.5 billion for their treatment [2]. These infections are a real threat to global public health and numerous efforts are underway to contain the spread of MDR strains, particularly in hospital settings and cities with high population [3]. A very recent example of a pandemic threat is represented by infections caused by Gram-positive methicillin resistant *Staphylococcus aureus* (MRSA) strains and vancomycin resistant enterococci (VRE). The first case of MRSA was identified in the early 60s and currently this infection appears at high incidence in Europe, Asia and America [4]. In the latter, MRSA infections provoke more deaths annually than AIDS, emphysema and homicides [5]. MRSA strains can be classified into hospital-acquired MRSA (HA-MRSA) and community-acquired MRSA (CA-MRSA), according to their original sources, but more recently, several MRSA strains resulted to be not strictly related to health care-associated infections, e.g., MRSA associated to livestock (LA-MRSA) [6,7,8]. The other challenge of public health is given by VRE infections. These are commonly caused by *Enterococcus faecium* and *Enterococcus faecalis* and provoke surgical-site, urinary tract and bloodstream infections [9]. Although with a lower incidence than MRSA, VRE cause about 66,000 infectious cases in the U.S. annually. Another example of dangerous infections is represented by carbapenem-resistant Enterobacteriaceae (CRE), a group of bacteria such as *Klebsiella pneumoniae* and *Escherichia coli*, which produce enzymes (e.g., New Delhi metallo-beta-lactamase, NMD-1) able to make them resistant to virtually all beta-lactams. As of February 2019, the US Food and Drug Administration (FDA) approved some antibiotic drugs i.e., ceftazidime-avibactam, meropenem-vaborbactam, plazomicin and eravacycline for treatment of some CRE-related infections [10]. In light of these difficult resistant infections and the scarcity of new approved antibiotics, it is now evident that research must open the horizons to new therapeutic strategies, new combinatorial therapies of drugs and to the discovery of new antimicrobials [11,12,13].

### The Need for New Antimicrobials

Antibiotics have drastically changed people’s lives: in America, in 1920, life expectancy was 56 years while in 2020 it was around 80; indeed, in developing countries, antibiotics have reduced the morbidity and mortality caused by food-borne and poverty-related diseases [8].

However, large pharmaceutical companies averaged a drastic decline in the production of new antibiotics [14]. This is because the economic crisis at the beginning of the century has led to substantial cuts in academic research and health care spending; in addition, pharmaceutical industries have been more oriented towards investment of drugs capable of curing chronic diseases, which translate into greater economic revenues. It is worthwhile noting that in America a chemotherapy treatment can cost tens of thousands of dollars, compared to about 3,000 of an antibiotic therapy [15,16]. In addition, the easy availability of antibiotics and the relatively low costs make them of little value to consumers. It follows that a new antibiotic drug should not cost much more, to be purchased. Finally, regulation for clinical trials has become much more complex: studies with antibiotics and placebo are now considered unethical, so trials can only be conducted to demonstrate better drug activity than an existing antibiotic. This results in longer and more expensive clinical trials [15]. Moreover, once on the market, the antibiotic may become useless by the appearance of resistance. As a consequence, new strategies and new sources of antimicrobial molecules are highly demanded.

Nature is undoubtedly the richest source of molecules with the most varied biological features. Due to its biodiversity not only between animal and plant kingdoms, but also among the various species, nature represents the largest library of compounds that has ever existed [17,18,19].

Many of these molecules exhibit antimicrobial activity and have a chemical structure often very different from each other. Examples are antimicrobial peptides produced by insects, amphibians, mammals and plants [12,20,21,22]. These can have a cyclic or a linear structure, and consist of no more than 50 amino acids and have various biological properties, from an antimicrobial to an immunomodulatory function [22,23,24,25,26]. Another promising class of natural compounds from the plant kingdom is given by secondary metabolites. Many of these (e.g., tannins, terpenes, carotenoids, polyphenols and alkaloids) have already been characterized for their biological properties and relevance as potential new antimicrobials [27,28,29,30,31]. Furthermore, these molecules can serve as a chemical scaffold for the synthesis of libraries in order to identify markers for a specific detection or for the design of lead compounds with a desired biological activity [32,33,34].

In this review, we focus on alkaloids derived from higher plants as potential new antimicrobials against antibiotic-resistant infections. As reported in Table 1 and Table 2, these compounds are already on the market or in clinical trials, confirming their valuable power for the development of new drugs for treatment of different types of diseases.

## 2. Alkaloids

**Figure 1:**
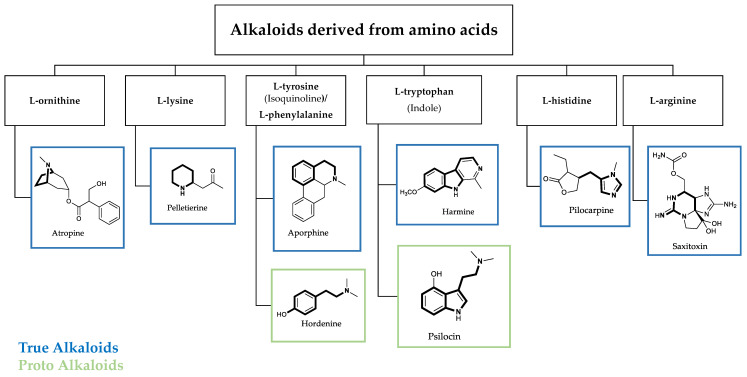
Schematic representation of true- and proto-alkaloids. The amino acidic skeleton derived from the natural precursor is in bold.

**Figure 2:**
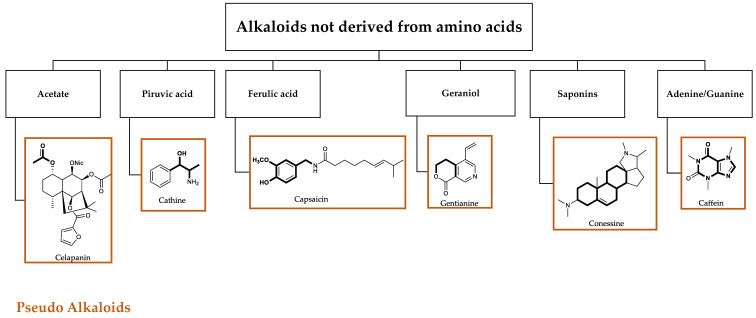
Schematic representation of pseudo-alkaloids. The carbon skeleton derived from the natural precursor is in bold.

Alkaloids represent a wide and structurally diverse group of secondary metabolites that can be found in 300 plant families, as well as in bacteria, fungi and animals [48]. To date, more than 18,000 different alkaloids have been discovered [49,50]. The name ‘alkaloid’ (alkali-like) is due to their basic nature, which allows them to be found as salts of organic acid or free bases. An individual alkaloid name consists of the permanent suffix ‘-ine’, linked to their amino origin, and by a more changeable prefix. This can be named after pharmacological activities (e.g., emetine), their discoverer (e.g., pelletrine) and the source’s geographic location from which they were isolated (e.g., atropine) [51,52]. Alkaloids are characterized by great structural diversity; the sole unifying feature is the presence of a basic nitrogen atom that can occur in the form of a primary amine (RNH_2_), a secondary amine (R_2_NH) or a tertiary amine (R_3_N) [51]. They can occur as monomers or they can form oligomers (homo or hetero-oligomers). Although there is no standard taxonomic classification, alkaloids can generally be classified according to their chemical structure, biochemical pathway or natural origin [53]. From a biosynthetically point of view, alkaloids can be divided into three major categories: true-, proto- and pseudo-alkaloids (Figure 1 and Figure 2).

**Scheme 1:**
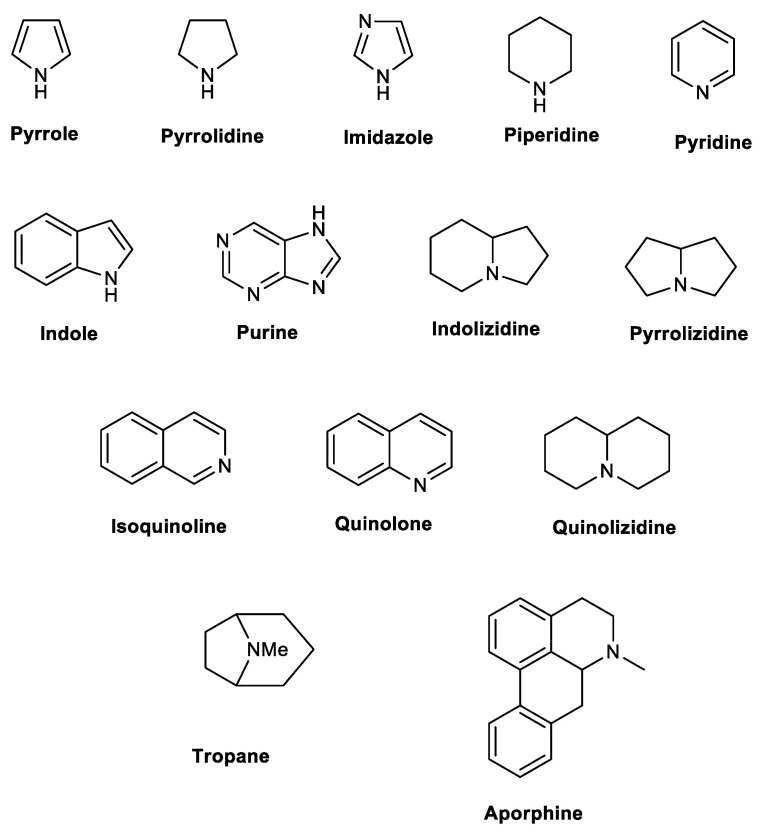
The 14 subgroups of alkaloids.

True- and proto-alkaloids have an amino acid as a precursor, but they differ for the presence or not of the N-atom in the heterocycle, respectively. Pseudoalkaloids feature a basic carbon skeleton not deriving from an amino acid [54]. Alkaloids are often classified on the basis of their chemical structure in heterocyclic or typical alkaloids (true alkaloids), containing nitrogen in the heterocycle, and non-heterocyclic or atypical (proto-alkaloids), containing nitrogen in a side chain [48]. Since their structural complexity and according to their backbone, heterocyclic alkaloids can be split into 14 subgroups including indoles, isoquinolines, pyrrolizidines, pyrrolidines, quinolizidines, tropanes, purines, piperidines and imidazoles (Scheme 1) [50].

Alkaloids have been extensively investigated for their biological activity (e.g., anticancer, antibacterial, antiviral and central nervous depressant activity) in both traditional and modern medicine [43]. Notably, their exceptional biological activity is provided by the ability to form hydrogen bonds with enzymes, receptors and proteins due to the presence of a proton accepting nitrogen atom and one or more protons donating amine hydrogen atoms. In recent years, the alkaloids’ antibacterial activity played a significant role in the treatment of many infectious diseases reporting MDR phenomena. This led researchers to direct their attention onto these promising plant secondary metabolites [55]. Thus, the development of different extraction methods to obtain pure alkaloids results to be very important, even if they are often produced in very small amounts by their natural source and their enantioselective separation is quite difficult, mostly due to the presence of a large number of chiral centers. In order to overcome these issues, a wide range of synthetic efforts has been recorded with the aim to achieve enantiomerically pure alkaloids [56]. One of the most direct, efficient, and variable synthetic methods for the construction of privileged pharmacophores (i.e., tetrahydro-isoquinolines, tetrahydro-β-carbolines and polyheterocyclic frameworks) and for the creation of natural compounds libraries in medicinal chemistry proved to be the Pictet-Spengler reaction [57,58]. This reaction, in combination with chiral catalysts, has been reported in the total synthesis of complex alkaloids [59]. Another synthetic approach widely employed for the construction of sophisticated macromolecules architecture, such as alkaloids, is the olefin metathesis reaction, which is one of the most powerful tools for the formation of challenge polycyclic frameworks and bridged nitrogen heterocycles [60,61,62]. Most of the alkaloids reported below are known and their multiple chiral centers were assigned according to the literature. The absolute configuration is not reported for alkaloids tested as the racemic form.

### 2.1. Indole Alkaloids

**Figure 3:**
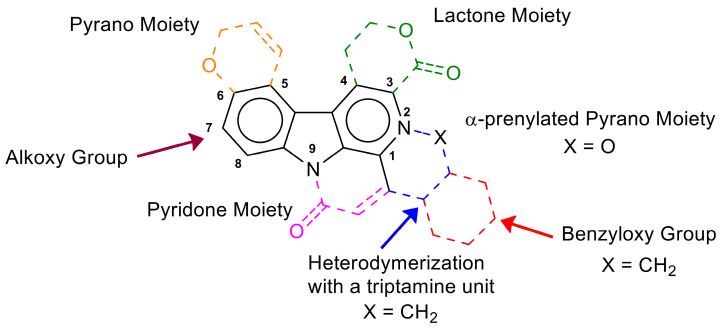
Beneficial modifications of the indole alkaloids-based skeleton.

Indole alkaloids may provide novel promising chemotypes for drug discovery due to their structural diversity. More than 4,000 known compounds, biosynthetically derived by l-tryptophan, are classified as indole alkaloids. This class of alkaloids shows a bicyclic structure formed by a benzene ring fused to a five-membered pyrrole ring and they differ in the presence of carbonyl, methoxyl and hydroxyl groups at different positions [63]. Indole alkaloids are not a homogenous group that can be classified according to different criteria. The main subclasses are carbazole and β-carbolines but according to botanical sources, they can be further distinguished into: *Strychnos* alkaloids, yohimbans, heteroyohimbans, *Vinca* alkaloids, β-carbolines, kratom alkaloids, tryptamines, ergolines or clavine alkaloids and *Tabernanthe iboga* alkaloids [64,65]. Some representative indole alkaloids discussed in this review are reported in Table 3 and the structure–activity relationships (SARs) analysis has been summarized in Figure 3.

#### 2.1.1. β-Carbolines

The β-carbolines consist of a tricyclic pyrido[3, 4-b] indole ring structure at different levels of unsaturation (dihydro-, tetrahydro and aromatic β-carbolines) and are classified according to their ABC skeleton as α-, β-, γ- and δ-carbolines. The α of carboline alkaloids is l–tryptophan, the β is tryptamine and the γ is dihydro-β-carboline, where the carboline nucleus is formed [77,78]. Interestingly, for β-carbolines a greater selectivity towards the human pathogen *S. aureus* has been observed. Darabpour et al. evaluated the antimicrobial effect of the *Peganum harmala* extracts against several MDR Gram-positive and Gram-negative clinical isolates and reported that the crude extract of *P. harmala* seeds and roots exhibited a good synergistic effect upon coadministration with novobiocin, carbenicillin and colistin [79]. Further investigations revealed that the seeds and the roots extract of *P. harmala* are a considerable source of β-carbolines such as harmaline, haman, harmalol and harmine [50]. Interestingly, Mothar et al. investigated the efflux pump inhibitor (EPI) activity of 13 antibacterial alkaloids, against a panel of three MRSA strains. Among them, harmaline (Table 3) was able to reduce by 4-8-fold the minimum inhibitory concentration (MIC) of ethidium bromide (EtBr), a popular efflux substrate for many efflux systems. The authors postulated that amongst the indole alkaloids the presence of a methoxy group at the C-6 position of the aromatic ring coupled with a secondary amine group in the pyrrole ring might affect the EPI activity [66]. Canthine-6-one type alkaloids, bearing an additional D ring (ABCD core), a pyridone, are well-known constituents of the Simaroubaceae and Rutaceae, and antibacterial activity is well described [78]. Interestingly, O’Donnell et al. investigated the antimicrobial activity of two canthine-6-one type alkaloids, canthin-6-one and 8-hydroxy-canthin-6-one (Table 3), isolated from *Allium neapolitanum*, which showed a potent activity against MRSA and MDR *S. aureus* with MIC values ranging from 8 to 64 μg/mL [67]. Recently, Casciaro et al. evaluated the antibacterial activity of 39 alkaloids available in a unique in-house library of about 1,000 natural compounds against a Gram-positive (*S. aureus* ATCC 25923) and a Gram-negative (*E. coli* ATCC 25922) reference bacterial strain [68]. Interestingly, a greater selectivity towards the human pathogen *S. aureus* was observed for the β-carboline alkaloids, especially for nigritanine (Table 3), a rare β-carboline heterodimer and some of its monomeric analogs (i.e., speciociliatine, mytragine and paynantheine). Further investigations confirmed nigritanine as a potent antistaphylococcal agent, with a remarkable activity against three MDR clinical isolates of *S. aureus* with an MIC value of 128 μM and a negligible cytotoxicity, features not observed for the other tested β-carboline analogues. Chemically, nigritanine is a heterodimer alkaloid formed by the union of a corynane and a tryptamine unit and isolated from different *African strichnos* species [80]. The analysis of the antibacterial activity related to the corynane scaffold provided new insights in the SARs of β-carboline, confirming that dimerization improves the antibacterial activity possibly because the larger molecule is less susceptible to bacterial efflux [68,78].

#### 2.1.2. Carbazoles

Carbazoles, a group of indole alkaloids featuring various structural features, are widely investigated for their anti-MDR activity [81]. *Clausena harmandiana* and *Clausena wallichii*, selected members of the genus *Clausena* belonging to the Rutaceae family, represent the most important sources of bioactive carbazole alkaloids [70,71]. An extensive investigation of biologically active natural products from *Clausena* plants led Maneerat et al. to identify three new carbazole alkaloids, harmandianamines A-C, together with fifteen known compounds, from the twigs of *C. harmandiana* [69]. All compounds, many of them were indole alkaloids, were tested against a panel of Gram-positive bacteria, including MRSA SK1, and Gram-negative bacteria. Two lactonic carbazole alkaloids, clausamine A and clausamine B (Table 3), featuring 1-oxygenated 3,4-disubstituted structures with a lactone moiety and a 4-prenylcarbazole alkaloid, clausine F (Table 3), showed a potent antibacterial activity against MRSA SK1. In particular, clausamine A and clausine F displayed MIC values of 8 and 4 μg/mL, respectively, but more interestingly clausamine B was found to be a potent antibacterial compound against MRSA SK1 with a MIC of 0.25 μg/mL, which is lower than that of commonly used vancomycin (MIC of 1 μg/mL) [69]. Furthermore, the authors evaluated the antimicrobial characterization of four new carbazoles, clausenawallines C, D, E and F, along with 18 known indole alkaloids isolated from the roots of *C. wallichii* [70]. Among all compounds tested, clausenawallines E (Table 3), an unsymmetrical pyranocarbazole-type heterodimer, and a 1-prenylcarbazole alkaloid, 2,7-dihydroxy-3-formyl-1-(3′-methyl-2′-butenyl)carbazole (Table 3) exhibited a strong antibacterial activity against MRSA SK1 with MIC of 8 μg/mL and 4 μg/mL, respectively [70]. As a continuation of their study, Maneerat et al. investigated the antimicrobial activity of five new carbazole alkaloids, clausenawallines G–K (Table 3), isolated from the twigs of *C. wallichii*. Unfortunately, all the new compounds had a weaker antibacterial activity against MRSA SK1 with MIC values ranging from 64 to 128 μg/mL [71]. Nevertheless, the studies of this research group provided new insights in the analysis of the anti-MRSA activity related to the carbazole scaffold, confirming the high potential of this versatile scaffold for the development of novel alkaloid derivatives with improved activity and suggested lactonic, prenilated and pyrano as ideal carbazole-type scaffolds for further chemical modifications. Notably, in the case of pyranocarbazole alkaloids heterodimerization seems to enhance anti-MRSA activity.

In a previous work, three prenylated pyranocarbazole alkaloids from the leaves of *Murraya koenigii* (Rutaceae) mahanine, mahanimbicine and mahanimbine were tested against five antibiotic resistant pathogenic bacteria. These alkaloids exhibited a moderate antibacterial activity with MICs ranging from 25.0 to 175.0 mg/mL but, more interestingly, mahanine (Table 3) exerted the strongest activity specifically against *Streptococcus pneumoniae* with a MIC value of 12.5 mg/mL suggesting that little chemical changes of pyranocarbazole scaffold can affect the activity [72].

#### 2.1.3. Yohimbans

Since the isolation of yohimbine by Spiegel in 1900 and its structural determination by Witkop in 1943, a broad variety of natural yohimbine stereoisomers have been identified [82]. This family of pentacyclic indole alkaloids, which originated from l-tryptophan and secoiridoid monoterpene secologanin, can be subdivided into four different subfamilies, which differ in the stereochemical arrangement around the d-ring. The most representative members are yohimbine, rauwolscine, pseudoyohimbine and reserpine, respectively normal, allo, pseudo or epiallo [83]. Reserpine is an indole alkaloid (Table 3), naturally occurring in *Rauwolfia serpentine*, which is known to be a competitive inhibitor of both primary and secondary active transporter systems [84,85,86,87,88,89,90,91,92]. In particular, regarding this latter function, reserpine acts mainly on resistance nodulation division (RND) and the major facilitator superfamily (MFS). Recently, Shaheen et al. reported a reserpine inhibitory effect on RND transporter Acriflavine resistance protein B (AcrB). A preliminary docking analysis of reserpine towards the *Salmonella typhi* AcrB protein structure showed that it shares its binding site with ciprofloxacin, a known substrate of AcrB, suggesting a possible activity of this alkaloid as a competitive inhibitor. Furthermore, *in vitro* studies were initially carried out by a disk diffusion assay and later by following time-dependent growth. The combination of reserpine with ciprofloxacin resulted in enhanced drug-induced growth inhibition of *E. coli* C41(DE3) cells expressing AcrB protein transporter. This study supported the role of reserpine in modulating AcrB activity and potentiating the ciprofloxacin action against host cells [93]. Interestingly, Tariq et al. reported the EPI activity of reserpine against the efflux transporter STY4874, belonging to MFS, overexpressed in pMR4/*E. coli*. Measurement of inhibition zones of pMR4/*E. coli* cells (with no expression of STY4874) indicated that cells were unaffected to the combination of reserpine and ciprofloxacin, whereas, growth of pMR4-STY4874/*E. coli* cells was strongly affected by the combination of ciprofloxacin and reserpine, resulting in larger zone of inhibition (19.0 ± 1.0 mm). The evidences of the disk diffusion assay were confirmed by other experiments suggesting that reserpine when tested in combination with ciprofloxacin exerted significant STY4874-mediated inhibitory activity leading to the accumulation of ciprofloxacin inside the cell. These results could be also extended towards STY4874 close homologues, MdfA and MdtM from *E. coli* making this study an important starting point to further develop potent inhibitors of these efflux pumps [73]. Nevertheless, the potential of reserpine as EPI limits its usage due to the toxic effect to humans at the concentration needed for inhibition.

#### 2.1.4. Clavine Alkaloids

Clavine alkaloids consist of a diverse group of chemical compounds with a structural skeleton deriving from the alkaloid ergoline, therefore they are also known as ‘Ergolines’. Several studies reported that most of the clavine alkaloids did not have antibacterial activity per se but behaved like co-adjuvants of antibiotics [74,94,95,96]. The seeds of *Ipomoea muricata* have been reported to contain about 0.49% clavine alkaloids, of which lysergol constitutes 53% and chanoclavine 37% [97]. In a previous investigation Maurya et al. optimize the isolation of lysergol and chanoclavine (Table 3) from this plant [98]. Furthermore, the authors evaluated the antibacterial activity of lysergol and thirteen acyl and aryl semi-synthetic derivatives and their synergy with the antibiotic nalidixic acid (NA) against nalidixic acid-sensitive (NASEC) and nalidixic acid-resistant (NAREC) strains of *E. coli* [74]. Lysergol (Table 3) did not possess antibacterial activity of its own, but in combination, it strongly reduced the MIC of nalidixic acid by 8-fold against the NAREC and 4-fold against the NASEC. Interestingly, two aryl semi-synthetic derivatives, 17-O-3″,4″,5″-trimethoxybenzoyllysergol and 17-O-3″-nitrobenzoyllysergol (Table 3), reduced the MIC of nalidixic acid by 8-fold against both NASEC and NAREC strains. Lysergol and these derivatives were also tested in combination with another antibiotic, tetracycline, against a multidrug-resistant clinical isolate of *E. coli* (MDREC) and showed significant synergistic activity, reducing the MIC of the drug by 8-fold [74]. Recently, the same research group investigated the synergy potential and the drug resistance reversal mechanism of chanoclavine and lysergol from *I. muricata* against the multi-drug-resistant clinical isolate of *E. coli* (MDREC) [75]. Chanoclavine (Table 3) showed the highest resistance reversal potential reducing the MIC of tetracycline by 8-16-fold, probably due to the higher aqueous solubility of secondary amine than the tertiary amine of lysergol. In real-time expression analysis, chanoclavine exhibited down-regulation of different efflux pump genes and decreased the mutation prevention concentration of tetracycline. *In silico* docking analysis of chanoclavine towards the receptor proteins AcrB, YojI and OmpX, known to be involved in drug resistance, showed strong binding affinity. These studies supported the role of the chanoclavine as an inhibitor of tetracycline efflux from MDREC [75]. Ponnusamy et al. investigated the EPI activity of indirubin (Table 3), a bisindole alkaloid, isolated from the leaves of *Wrightia tinctorial*, using the NorA hyperexpression *S. aureus* SA1199B, and its synergistic effects were tested with ciprofloxacin [76,99]. Indirubin exerted antibacterial activity against both *S. aureus* SA1199B and the parent *S. aureus* SA1199, with MICs of 1.25 μg/mL and 25 μg/mL, respectively. More interestingly, these alkaloids synergistically enhanced the activity of ciprofloxacin by reducing 4-fold its MIC and the agar diffusion study showed an elevated ciprofloxacin inhibition zone in SA1199B by the addition of indirubin (2.5 and 1.25 μg/mL) suggesting its ability to block the NorA efflux pump followed by the increasing concentration of ciprofloxacin within the cell. These results suggested that this natural compound could be used in future therapeutic applications as a potential EPI [76,99].

### 2.2. Isoquinoline Alkaloids

**Figure 4:**
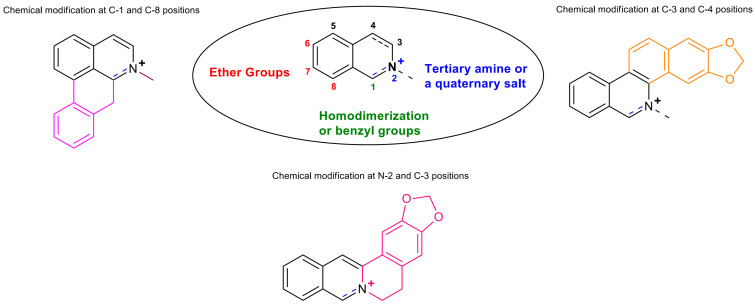
Beneficial modifications of the isoquinoline alkaloids based skeleton.

Isoquinoline alkaloids are a heterogeneous group biogenetically derived from l-phenylalanine and l-tyrosine and featured an isoquinoline or a tetrahydroisoquinoline skeleton. Based on their distribution, intramolecular rearrangements and the presence of additional rings linked to the main system, they can be classified into eight subgroups: protoberberine, benzo[c]phenanthridine, benzylisoquinoline, aporphine, protopine, phthalideisoquinoline, morphinan and emetine alkaloids [65]. Some representative isoquinoline alkaloids along with their antimicrobial effect are reported in Table 4 and the SARs analysis has been summarized in Figure 4.

#### 2.2.1. Protoberberines

Protoberberines are the largest group of isoquinoline alkaloids, which makes them the most widespread secondary metabolites containing nitrogen. Berberine (Table 4) is the main representative quaternary ammonium salt of protoberberine’s class produced from *Berberis* spp. Numerous investigations described a moderate antimicrobial activity of berberine, especially against Gram-negative bacteria. This is probably due to its similarity with the substrate of the multi-drug resistance efflux pumps [112,113,114,115]. Accordingly, the presence of MDR pump inhibitors remarkably increases the susceptibility of bacterial strains. Yu et al. studied the antibacterial activity of berberine and the synergy with β-lactam antibiotics against several MRSA clinical isolates and the standard MSSA. Berberine displayed antimicrobial activity against all tested MRSA strains with MIC values ranging from 32 to 128 μg/mL, and a MIC of 128 μg/mL against the standard MSSA strain. Interestingly, berberine strongly reduced the MICs of ampicillin and oxacillin against MRSA and a standard MSSA. The fractional inhibitory concentration index (FICI) of berberine in combination with ampicillin and oxacillin was 0.625 and 0.5 in MRSA, respectively. These data suggested an additive effect for the first combination, and a synergistic effect for the second one. The authors postulated that the synergistic activity of berberine and β-lactam antibiotics might be due to a different mode of action of berberine, which could bind to minor groves of DNA and regulate the gene expression [100]. Despite the antimicrobial activity of berberine, the mechanism of action in bacteria has remained unclear. Several studies demonstrated that berberine is a DNA-intercalating compound, however, recent data have supported the hypothesis that inhibition of bacteria’s cellular division protein FtsZ is the primary mechanism of its antibacterial activity [114,115,116,117,118]. Numerous scientists studied the effects of berberine on RND efflux systems inhibition in *P. aeruginosa* and, recently, it was reported as a potential inhibitor of MexXY-mediated resistance in this strain. Su et al. evaluated the efficacy of berberine alone and in combination with imipenem against a clinical *P. aeruginosa* isolate (PA012) and the possible EPI mechanism. The combination of berberine (1/4 MIC) with imipenem (1/8 MIC) exhibited a synergistic effect with a FICI of 0.375. Further investigations confirmed that berberine displayed a synergistic effect with the carbapenem antibiotic to resensitize imipinem-resistant *P. aeruginosa* via inhibition of the MexXY-OprM efflux pump system [101,112,119,120,121]. Furthermore, Laudadio et al. developed an *in silico* protocol to evaluate the putative ability of berberine to counteract the activity of the aminoglycoside extruder pump MexXY-OprM. Interestingly, molecular docking analysis showed that the aminoglycoside tobramycin and berberine competed for the same site but the MexY−berberine complex showed a much lower free energy. These results indicated that the berberine has a higher binding affinity than the tobramycin suggesting that it acts as a competitor of the antibiotic, preventing its extrusion. *In vitro* assays demonstrated a significant reduction (16-fold, from 16 to 1 μg/mL) of the tobramycin MIC in combination with berberine against *P. aeruginosa* strain C25, a CF isolate selected because of its MexY overexpression and a lack of acquired tobramycin resistance genes, and a comparable synergistic activity was confirmed by the results obtained with 12 additional *P. aeruginosa* clinical isolates [101].

#### 2.2.2. Benzophenanthredines

Sanguinarine (Table 4) is a benzophenanthridine alkaloid derived from the roots of *Sanguinaria canadensis* [122] structurally related to berberine. For this reason, it can be assumed that it presents a similar antibacterial activity by the inhibition of the Z-ring formation on MRSA, MSSA vancomycin-sensitive (VSE) and vancomycin-resistant strains (VRE) of *E. faecalis* [123,124,125,126] and by the intercalation with bacterial DNA [127,128,129]. Hamoud et al. investigated the antimicrobial activity of individual drugs, e.g., the DNA intercalating sanguinarine, the chelator ethylenediaminetetraacetic acid (EDTA) and the antibiotic streptomycin; of two-drugs interaction between EDTA or antibiotics and sanguinarine in comparison with the three-drug activities against several Gram-positive and Gram-negative bacteria, including multi-resistant clinical isolates [102,127]. Among the three drugs, sanguinarine demonstrated the strongest antibacterial activity against Gram-positive bacteria with MIC values ranging between 0.5 μg/mL against *S. epidermidis* and 8 μg/mL against VRE, whereas streptomycin showed the strongest activity against Gram-negative strains. EDTA showed only bacteriostatic activity. Interestingly, the three-drug combination displayed synergistic activity against almost all the strains (except methicillin- resistant *S. aureus*), as well as a strong reduction (2–16 times) in the effective doses (i.e., MIC of drug alone/MIC drug in combination) of sanguinarine, EDTA and streptomycin. The authors postulated that the synergistic interactions are due to different modes of action of the individual drugs: EDTA as a chelating agent disturbs the permeability of the bacteria cell wall leading to a higher influx of sanguinarine and streptomycin into the bacterial cell [102,127]. Choi et al. reported that a structural homolog of sanguinarine, the 6-methoxydihydrosanguinarine (Table 4), isolated from *Hylomecon hylomeconoides,* displayed an antibacterial activity higher than that of the antibiotic ampicillin against *S. aureus* ATCC 25923 (MSSA), *S. aureus* ATCC 33591 (MRSA) and DPS-1 (clinical MRSA) strains with MICs in the range of 1.9–3.9 μg/mL. These promising results indicated the benzophenanthridine alkaloid sanguinarine as a potential agent against MRSA strains paving the way for further studies [103]. Several studies reported a significant antibacterial activity of chelerythrine (Table 4), a benzophenanthridine alkaloid structurally related to sanguinarine, especially against Gram-positive bacteria [130,131]. Recently, He et al. investigated the antibacterial effect and mechanism of action of chelerythrine isolated from *Toddalia asiatica* (Linn) Lam widely used in traditional Chinese medicine. Interestingly, chelerythrine displayed a strong antibacterial activity against *Staphylococcus aureus* (SA), methicillin-resistant *S. aureus* (MRSA) and extended spectrum β-lactamase *S. aureus* (ESBLs-SA) with MIC values of 156 μg/mL. Further investigations on the anti-bacterial mechanism indicated that chelerythrine may be capable of destroying the channels across the bacterial cell membranes, leading to protein leakage to the outside of the cell, and to the inhibition of the protein biosynthesis. Images of scanning electron microscope revealed important morphological changes in chelerythrine-treated bacteria providing new insights in the antibacterial mechanism of this alkaloid [104]. Extensive investigation of the antimicrobial activity of natural products from *Zanthoxylum* genus, which represents the most important source of bioactive benzophenanthridine alkaloids, was carried out. Rodriguez et al. evaluated the antibacterial activity against MRSA of several compounds, isolated from *Zanthoxylum monophylum* widely used in Brazilian traditional medicine for the treatment of different health problems. Among them, two chelerythrine analogs, bis-[6-(5,6-dihydro-chelerythrinyl)]ether and 6-ethoxy-chelerythrine (Table 4), displayed a strong activity against MRSA with IC50 values of 1.0, and 4.0 μM, respectively [105]. Furthermore, Costa et al. reported the anti-MRSA activity of dihydrochelerythrine (Table 4) and *N*-methylcanadine (Table 4) against four MRSA clinical isolates with MICs ranging from 85.8 to 171.7 μM and from 76.9 to 307.8 μM, respectively [106]. In a previous work, Zuo et al. isolated the other three benzophenanthridine alkaloids, 6-hydroxydihydrosanguinarine, 6-hydroxydihydrochelerythrine and dihydrosanguinarine (Table 4), from *Chelidonium majus* Linn., along with dihydrochelerythrine, and investigated their antibacterial activity against twenty clinical strains of MRSA. The two non-hydroxylated benzophenanthridine alkaloids exhibited moderate or no inhibitory effects at the tested maximum concentration of 3,000 μg/mL, whereas 6-hydroxydihydrosanguinarine and 6-hydroxydihydrochelerythrine reported MICs/minimal bactericidal concentration (MBCs) values against MRSA strains as low as to 0.49/1.95 and 0.98/7.81 μg/mL, respectively [107]. These evidences supported the great potential of the benzophenanthridine alkaloid scaffold for the further development of derivatives with improved activity. Moreover, several structure–antimicrobial activity relationship studies of benzophenanthridine alkaloids structurally related to sanguinarine and chelerythrine were carried out [132,133]. Miao et al. evaluated the antibacterial activity of a series of alkoxyl and acetonyl derivatives at position 6 of sanguinarine and chelerythrine and postulated that the double bond of C=N^+^ was essential. This hypothesis was confirmed by Tavares et al., who observed that the nitrogen ring substituted with a methyl group, or in the form of a tertiary amine or a quaternary salt, is fundamental for antimicrobial activity. These investigations also suggested that a methylenedioxy group at C-7 and C-8, such as in sanguinarine, was responsible for a broader antibacterial spectrum than methoxyl groups at C-7 and C-8, such as in chelerythrine [132]. A recent study, reported by Khin et al., supported SAR studies. The authors evaluated the antimicrobial activity of sanguinarine and chelerythrine, isolated from *Macleaya cordata* (Chinese plume poppy), against wild-type, methicillin-resistant and multiple-resistant strains of *S. aureus* (SA1199, AH1263 and IA116, respectively). The two benzophenanthridine alkaloids exhibited a strong antibacterial activity against all strains of *S. aureus* with MICs ranging from 3 to 10 μg/mL, confirming the essential role of the double bond between carbon and positively-charged nitrogen species and methoxyl- and methylenedioxy- substitutions at positions C-7 and C-8 for the antibacterial activity [134].

#### 2.2.3. Bisbenzylisoquinolines

Tetrandrine and fangchinoline (Table 4) are two bisbenzylisoquinoline alkaloids isolated from the Chinese drug *Stephania tetrandra*. Zuo et al. evaluated the antimicrobial activity of these alkaloids and their synergy potential with antibiotics ampicillin, azithromycin, cefazolin and levofloxacin against ten clinical isolates of staphylococcal chromosomal cassette mec (SCCmec) III type methicillin-resistant *S. aureus* (MRSA). The two bisbenzylisoquinoline alkaloids displayed a good anti-MRSA activity with MIC/MBC values ranging from 64 to 128 μg/mL and from 256 to 1,024 μg/mL. Interestingly, a significant synergistic/additive antibacterial activity against 90% of the isolates was observed for the tetrandrine/cefazolin combination (FICIs ranged from 0.188 to 0.625) [108]. Furthermore, Fu et al. investigated the inhibitory effect of tetrandrine and fangchinoline against MRSA 13366 and ESBL-producing *E. coli* 13025. The results confirmed the potent antibacterial activity of tetrandrine, which exhibited MIC values of 80 and 160 μg/mL against MRSA and ESBL producing *E. coli*, respectively, whereas fangchinoline showed a moderated activity with MICs of 160 and 320 μg/mL [109]. Interestingly, these data indicated that a little chemical modification of tetrandrine scaffold, such as a hydroxy group at position 7 in fangchinoline leads to a lower antibacterial activity.

#### 2.2.4. Aporphines

Roemerine (Table 4) is an aporphine alkaloid isolated from several plants (*Annona senegalensi, Turkish Papaver* and *Rollinialeptopetala),* and previously reported for its activity against MDR bacteria. Yin et al. confirmed its effectiveness *in vitro* against four *S. aureus* strains (with MIC values ranging from 32 to 64 μg/mL) as well as *in vivo* against MRSA insepticemic BALB/c mice, and investigated the underlying mechanism indicating that roemerine increases cell membrane permeability in a concentration-dependent manner [110]. Recently, Akbulut et al. investigated roemerine as potential efflux pump inhibitor. The authors demonstrated that in *B. subtilis,* two MDR pumps Bmr (MFS transporter) and BmrA (ABC transports) were inhibited by this alkaloid. Several assays showed that roemerine potentiated the effect of berberine with MIC values reduced from 256 and 64 μg/mL and from 64 to 16 μg/mL, respectively, by inhibiting the Bmr efflux pump. In addition, transport assays conducted using *E. coli* inverted membrane vesicles overexpressing BmrA confirmed that increasing concentrations of roemerine inhibited the transport of the BmrA substrate, doxorubicin and through this pump [111].

### 2.3. Piperidines

A further subclass of alkaloids, used for the treatment of MDR infections, is piperidines. Biosynthetically, they are mostly derived from l-lysine and are characterized by a saturated piperidine ring. The most significant from a pharmacological standpoint was piperine (Table 5), the major constituent of black pepper (*Piper nigrum)* and long pepper (*Piper longum).* It is well known that this alkaloid is able to inhibit several cytochrome P450-mediated pathways and human P-glycoprotein [135,136], however, several studies reported the efflux inhibitory activity as a primary antibacterial mechanism against *S. aureus* and MRSA [66,95,137,138]. As previously described, Mothar et al. investigated antibacterial activity of several alkaloids and, except berberine, any inhibition was detected even at 250 µg/mL, suggesting these alkaloids as candidates for an EPI evaluation assay. Piperine was one of the alkaloids that exhibited notable potential EPI activities with a 8-fold EtBr MIC reduction against N441, in addition to a 4-fold EtBr MIC reduction against U949 and ATCC 25923, respectively. The authors postulated that the highly conjugated diene linked to the aromatic ring is essential for its EPI activity [66]. Khameneh et al. evaluated the synergistic antibacterial activity of gentamicin and piperine against MRSA and underlying mechanism of modulating bacterial resistance of piperine. The suitable way to administer this combination was via liposomal formulation, due to the antibiotics’ hydrophilicity and hydrophobicity of the alkaloid. Interestingly, the MIC value of gentamicin in the liposomal combination was reduced 32-fold when compared with the free respective drug showing a higher antibacterial activity in comparison with that of vancomycin. The authors also reported that accumulation study results indicated that percentages of entrapped ethidium bromide in the presence of piperine in both forms were increased supporting its potential role as pump efflux inhibitors. These findings suggested that piperine could enhance the antibacterial activity of gentamicin by inhibiting the efflux of the antibiotic [55].

### 2.4. Other Alkaloids (Quinolone and Indoloquinazolines)

Several studies reported the anti-MRSA activity of the extracts from *Tetradium ruticarpum* (‘Fructus Euodiae’), which is a considerable source of alkaloids [139,140,141,142]. Pan et al. investigated the anti-MRSA activity of six novel quinolones featuring aliphatic side chains at C-2 position, and, four of them exhibited activity against both the MRSA and standard strains with MIC values of 8 and 128 µg/mL, respectively. Among them, evocarpine (Table 5) showed the highest activity with MIC value of 8 µg/mL, 16-fold more active than oxacillin against MRSA, suggesting the role of 13-carbon monounsaturated aliphatic side chain in the antibacterial activity. These findings furnished new insights in the SAR of quinoline alkaloids [139]. Furthermore, Hochfellner et al. evaluated the antimycobacterial and modulating activity of evocarpine and two indoloquinazoline alkaloids, evodiamine (Table 5) and rutaecarpine (Table 5), isolated from *Fructus Euodiae*, against three MDR clinical isolates of *Mycobacterium tuberculosis*. Evocarpine was the most active compound against the MDR strains with MIC values ranging from 5 to 20 μg/mL and, more interestingly, in combination with the two indoloquinazoline alkaloid the growth inhibitory properties of the quinolone alkaloid were markedly attenuated. The authors postulated that the structural similarities between these alkaloids, leading to potential competition on the evocarpine molecular target, preventing these alkaloids from effectively disrupting the target protein [140]. One potential mechanism of action of these last alkaloids could be the inhibition of ATP-dependent MurE ligase of *M. tuberculosis*, enzyme involved in the biosynthesis of peptidoglycan principal constituent of the bacterial cell wall, but further studies are required [143].

**Table 5:** Summary of antimicrobial activity of some classes of piperidine and quinolone alkaloids.

Common Name	Chemical Structure	Tested Microorganism	Antimicrobial Effect	Source	Ref.
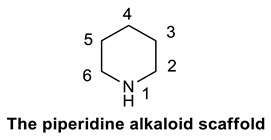					
Piperine	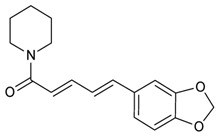	MRSA	Efflux Pump and cytochrome P450-mediated pathways Inhibitor	Species: *Piper nigrum Piper longum*	[55]
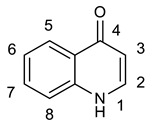					
Evocarpine	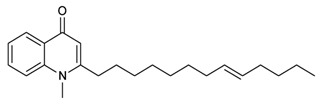	MRSA *M. tubercolosis*	Peptidoglycan biosynthesis Inhibitor	Species: *Tetradium ruticarpum*	[139,140]
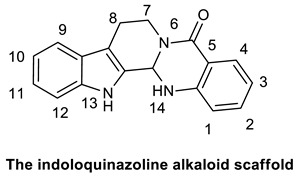					
Evodiamine	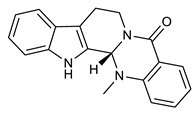	*M. tubercolosis*	Peptidoglycan biosynthesis Inhibitor	Species: *Tetradium ruticarpum*	[140,143]
Rutaecarpine	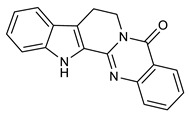	*M. tubercolosis*	Peptidoglycan biosynthesis Inhibitor	Species: *Tetradium ruticarpum*	[140,143]

## 3. Conclusions

The drastic drop in the number of new antibiotics on the market has led scientific research to reassess nature as an invaluable source of biologically active compounds. Among these, alkaloids of plant origin represent an interesting example of compounds for their biological and chemical properties. In this review we highlighted the potential of these alkaloids as antimicrobials specifically against strains resistant to conventional antibiotics or as adjuvants to be used in combination. The various data reported here have clearly shown that alkaloids can also be used as chemical scaffolds for further structural modifications. Taken all together, the data collected in this manuscript reinforce the idea that alkaloids can be considered as new alternative antimicrobials.

## Figures and Tables

**Table 1:** Plant-derived alkaloids in clinical trials (CT). Includes not yet recruiting, recruiting, enrolling by invitation, active not recruiting, suspended and completed trials of these compounds, parent extracts or derivatives, applied alone or as a mixture with other constituents.

Compound	Plant Source	Number of CT	Medicinal Purposes	Ref.
Atropine (tropane)	*Atropa belladonna, Hyoscyamus* spp., *Datura* spp.	241	Myopia, Refractive Errors, Bradycardia, Arrhythmias, Ventilator-Associated Pneumonia, Nausea, Vomiting, Cataract, Mydriasis, Spinal Anesthesia, Anesthesia, Anesthesiology Management, Postoperative Nausea, Hypotension, Hypotension After Spinal Anesthesia, Cesarean Section Complications, Endotracheal Intubation Amblyopia, Sialorrhea, Organophosphorus Poisoning.	[35,36]
Berberine (isoquinoline)	*Berberis* spp.	50	Colorectal Adenomas, Metabolic Syndrome, Schizophrenia, Coronary Artery Disease, Percutaneous Coronary Intervention, Ulcerative Colitis, Diabetes Mellitus, Chronic Kidney Disease, Gastritis, Peptic Ulcer, Dyspepsia, Type 2 Diabetes, Hypercholesterolemia, Nonalcoholic Fatty Liver Disease, Dyslipidemias, Prediabetes.	[37,38]
Camptothecin (indole)	*Camptotheca acuminata*	104	Malignant Lymphoma of Extranodal and/or Solid Organ Site, Solid Tumor, Lymphomas, Lung Diseases, Cancer, Corpus Uteri, Urothelial Carcinoma, Endometrial Cancer, Colorectal Cancer, Adenocarcinoma of the Esophagus, Adenocarcinoma of the Gastroesophageal Junction, Diffuse Adenocarcinoma of the Stomach, Malignant Glioma, Breast Cancer, Gastric Cancer, Lung Cancer, Metastatic Cancer.	[36,37]
Capsaicin (pseudo-alkaloid)	*Capsicum annuum* L. or *C. minimum* Mill	224	Pain, Neuropathic Pain, Cough, Cannabinoid Hyperemesis Syndrome, Spinal Cord Injuries, Sickle Cell Disease, Nonallergic Irritant Rhinitis, Pulmonary Hypertension, Irritable Bowel Syndrome, Colonic Diseases, Dyspepsia, Knee Osteoarthritis, Chemotherapy-induced Peripheral Neuropathy, Diabetic Nerve Problems, Diabetic Neuropathy, Diabetic Complications Neurological, Obesity, Insulin Resistance, Bronchiectasis, Rhinitis, Peripheral Nerve Injury, Postherpetic Neuralgia, Asthma, Migraine, Cluster Headache, Headache Disorders, Trigeminal Autonomic Cephalgia, HIV Infections, Peripheral Nervous System Diseases, Herpes Zoster, Alopecia Areata.	[39,40]
Colchicine (pseudo-alkaloid)	*Colchicum autumnale*	140	Coronavirus Infections, Corona Virus Disease 19 (COVID 19), Essential Hypertension, Heart Diseases, Atrial Fibrillation, Cardiac Surgery, Colchicine Adverse Reaction, Colchicine Resistance, Colchicine Toxicity, Pericardial Effusion, Chagas Disease, Arrhythmia, Acute Myocardial Infarction, Coronary Artery Disease, Acute Coronary Syndrome, Atherosclerosis, Inflammation, Diabetes, Hypertriglyceridemia, Gout, Pericarditis, Stroke, Myocardium Injury, Myocardial Infarction, Myocardial Ischemia, Familial Mediterranean Fever, Cholangiocarcinoma, Gout Flare, Pneumonia Viral, Arthritis Rheumatoid, Chondrocalcinosis, Osteoarthritis, Diabetes Mellitus Type 2, Colchicine mechanism of action.	[41]
Galantamine or Galanthamine (isoquinoline)	*Galanthus woronowii*, *Galanthus nivalis*, *Galanthus caucasicus* (Baker) Grossh.	97	Nicotine Addiction, Alzheimer Disease, Smoking, Schizophrenia, Major Depression, Bipolar Depression, Aphasia, Stroke, Cocaine Dependence, Dementia, Cognitive Impairment, Neurocognitive Disorders, Autism, Mental Disorders.	[36,40]
Papaverine (isoquinoline)	*Papaver somniferum* L.	17	Kidney Cancer, Pediatrics Anesthesia and Vasospasm, Lung Non-Small Cell Carcinoma, Radial Artery Injury Prevention, Prostatic Hyperplasia, Prostate Cancer, Injury of Internal Mammary Artery, Complications Due to Coronary Artery Bypass Graft, Erectile Dysfunction.	[42]
Piperine (piperidine)	*Piper nigrum*, *Piper longum*	18	Bladder Spasm, Malignant Neoplasm, Pain, Urinary Urgency, Deglutition Disorders, Chronic Kidney Diseases, Obesity.	[37,43]
Quinine (quinolone)	*Cinchona* spp.	67	Obesity, Plasmodium Falciparum Malaria, Malaria, Severe Malaria, Anemia, Cocaine Use, Pharmacokinetics, HIV Infections.	[35,36]
Reserpine (indole)	*Rauwolfia* spp.	9	Refractory Hypertension, Cocaine-Related Disorders, Substance-Related Disorders, Cardiovascular Diseases, Cerebrovascular Disorders, Heart Diseases, Hypertension, Schizophrenia, Parkinson’s Disease, Atherosclerosis, Hypercholesterolemia.	[35,36,37]
Solamargine (steroidal glycoalkaloid)	*Solanum* spp.	3	Actinic Keratosis.	[40,44]
Tetrandrine (isoquinoline)	*Stephania tetrandra*	1	Corona Virus Disease 2019, COVID-19.	[43]
Vincristine (indole)	*Catharanthus roseus* (L.) G. Don	885	Kaposiform Hemangioendothelioma, Kasabach-Merritt Syndrome, Tufted Angioma, Sarcoma, Neuroblastoma, Acute Lymphoblastic Leukemia, Rhabdomyosarcoma, Vincristine Induced Peripheral Neuropathy, B Cell Lymphoma, Lymphoma, Leukemia, Hematologic Diseases, Medulloblastoma, Recurrent Adult Burkitt Lymphoma, Low Grade Glioma, Metastatic Malignant Uveal Melanoma, Multiple Myeloma and Plasma Cell Neoplasm, HIV-1 Infection, Diffuse Astrocytoma, Anaplastic Astrocytoma, Astrocytoma, Sarcoma Kaposi, Ewing Sarcoma, Wilms Tumor, AIDS-Related Lymphoma, Brain and Central Nervous System Tumors.	[37,42]
Yohimbine (indole)	*Rauwolfia serpentine*	39	Parkinson Disease, Type 2 Diabetes, Erectile Dysfunction, Social Anxiety Disorder, Phobic Disorders, Post-Traumatic Stress Disorder, Involutional Depression, Major Depression, Opioid Use Disorder.	[45]

Determined from www.clinicaltrials.gov on 9 July 2020. Further information for alkaloids’ classification derives from: https://www.genome.jp/kegg-bin/get_htext?br08003.keg.

**Table 2:** Plant-derived alkaloids approved for therapeutic use.

Compound	Trade Name	Plant Source	Medicinal Purposes	Ref.
Atropine (tropane)	Atropen	*Atropa belladonna*, *Hyoscyamus* spp., *Datura* spp.	Spasmolytic agent for gastrointestinal tract, Pupil enlargement in eye.	
Caffeine (purine)	Cafcit, Vivarin, Alert	*Coffea arabica*, *Thea sinensis*	Treatment of apnea of prematurity and bronchopulmonary dysplasia in infants, Central nervous system stimulant.	[35]
Capsaicin (pseudo-alkaloid)	Qutenza	*Capsicum annum* L. or *C. minimum* Mill	Postherpetic neuralgia.	[40]
Codeine (isoquinoline)	Tuzistra XR^®^	*Papaver somniferum* L.	Analgesic, antidiarrheal and antitussive activity.	[36,42]
Colchicine (pseudo-alkaloid)	Colcrys, Mitigare	*Colchicum autumnale* L.	Gout, Familial Mediterranean Fever.	[40,42]
Ephedrine (pseudo-alkaloid)	Primatene, Bronkaid	*Ephedra* spp.	Treatment of asthma, hay fever, narcolepsy and depression.	[35]
Galantamine or Galanthamine (isoquinoline)	Reminyl^®^, Razadyne^®^, Nivalin^®^	*Galanthus woronowii, Galanthus nivalis, Galanthus caucasicus* (Baker) Grossh.	Treatment of dementia caused by Alzheimer’s disease and other central nervous system disorders.	[36,40,42]
Morphine (isoquinoline)	Statex, Oramorph, Sevredol, MS Contin	*Papaver somniferum* L.	Analgesic activity, management of chronic, moderate to severe pain.	[36,46]
Nicotine (pyridine)	Nicorette, Nicotrol	*Nicotiana tabacum* L.	Help for smoking cessation.	[36]
Omacetaxine mepesuccinate or Homoharringtonine (isoquinoline)	Synribo, Ceflatonin^®^	*Cephalotaxus harringtonia* (Knight ex Forbes) K. Koch	Oncology, Chronic myeloid leukemia.	[40,47]
Pilocarpine (imidazole)	Isopto Carpine, Salagen	*Pilocarpus jaborandi* Holmes	Treatment of Glaucoma, xerostomia and Sjogren’s syndrome.	[35,46]
Quinine (quinolone)	Qualaquin, Quinate, Quinbisul	*Cinchona* spp.	Antimalarial drug.	[35,36]
Reserpine (indole)	Raudixin, Serpalan, Serpasil	*Rauwolfia* spp.	Antihypertensive and antipsychotic.	[35,36,37]
Scopolamine (tropane)	Transderm Scop, Kwells, Buscopan	*Atropa belladonna, Hyoscyamus* spp., *Datura* spp.	Antiemetic, anticholinergic and spasmolytic agent.	[35,36]
Solamargine (steroidal glycoalkaloid)	Curaderm	*Solanum* spp.	Cancer chemotherapy.	[40,47]
Vinblastine (indole)	Velban, Alkaban-AQ^®^	*Catharanthus roseus* (L.) G. Don	Chemotherapy medication for several types of cancer.	[35,36]
Vincristine (indole)	Oncovin, Vincasar, Marqibo	*Catharanthus roseus* (L.) G. Don	Antineoplastic agent to treat various cancers.	[37,42]

Additional resources from https://www.drugs.com/ and https://www.drugbank.ca/; for alkaloids’ classification from: https://www.genome.jp/kegg-bin/get_htext?br08003.keg.

**Table 3:** Chemical structure and antimicrobial activity of some representative indole alkaloids.

Common Name	Chemical Structure	Tested Microorganism	Antimicrobial Effect	Source	Ref.
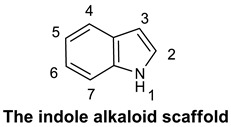					
**β-Carbolines**					
Harmaline	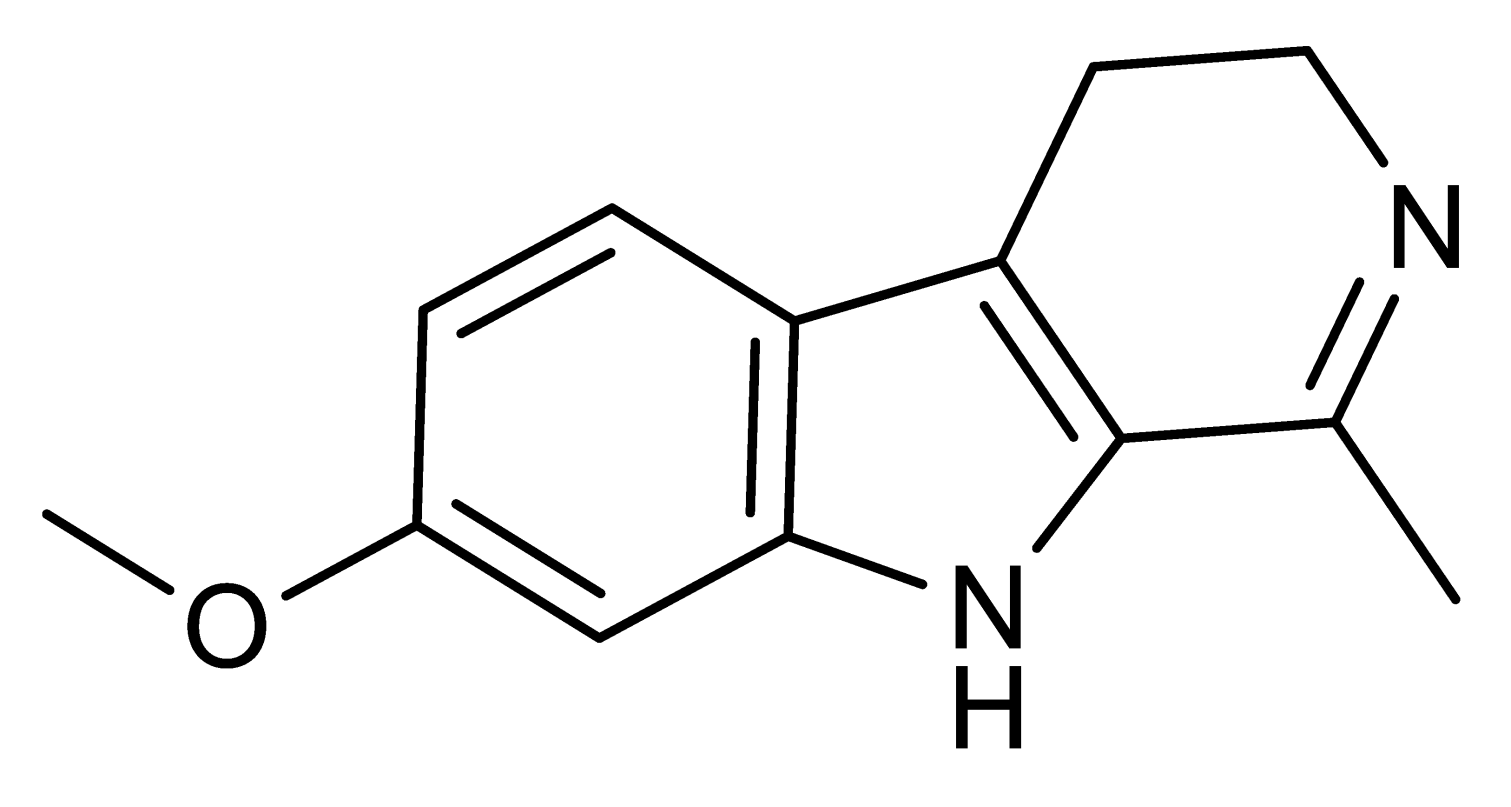	MRSA	Efflux Pump Inhibitor	Species: *Peganum harmala*	[66]
Canthin-6-one	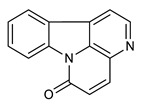	MRSA *S. aureus*	Growth inhibition	Species: *Allium neapolitanum*	[67]
8-Hydroxy-canthin-6-one	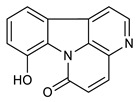	MRSA *S. aureus*	Growth inhibition	Species: *Allium neapolitanum*	[67]
Nigritanine	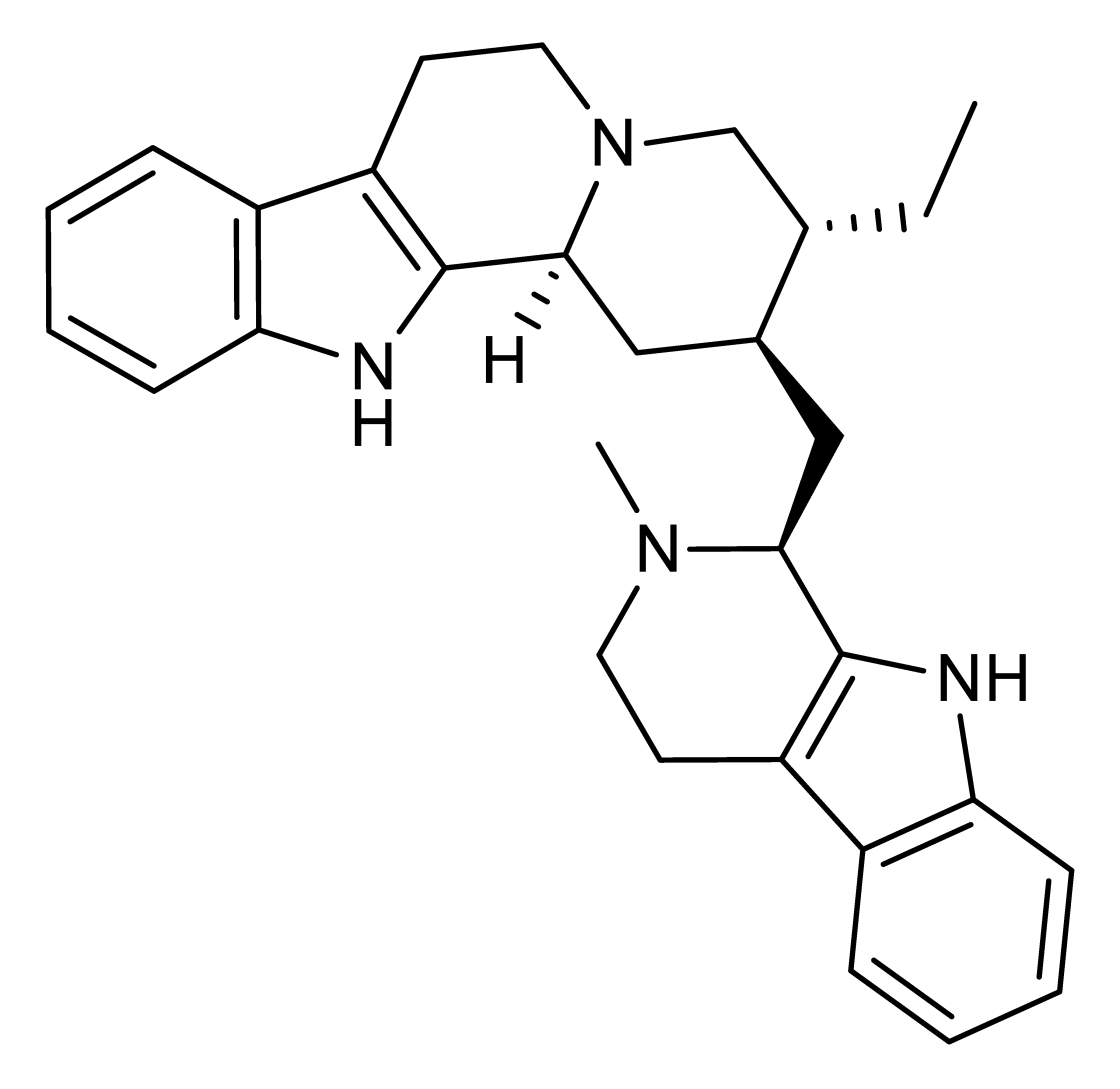	*S. aureus*	Growth inhibition	Species: *African Strichnos*	[68]
**Carbazoles**					
Clausamine A	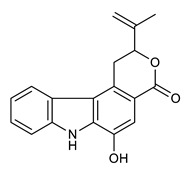	MRSA SK1	Growth inhibition	Species: *Clausena harmandiana*	[69]
Clausamine B	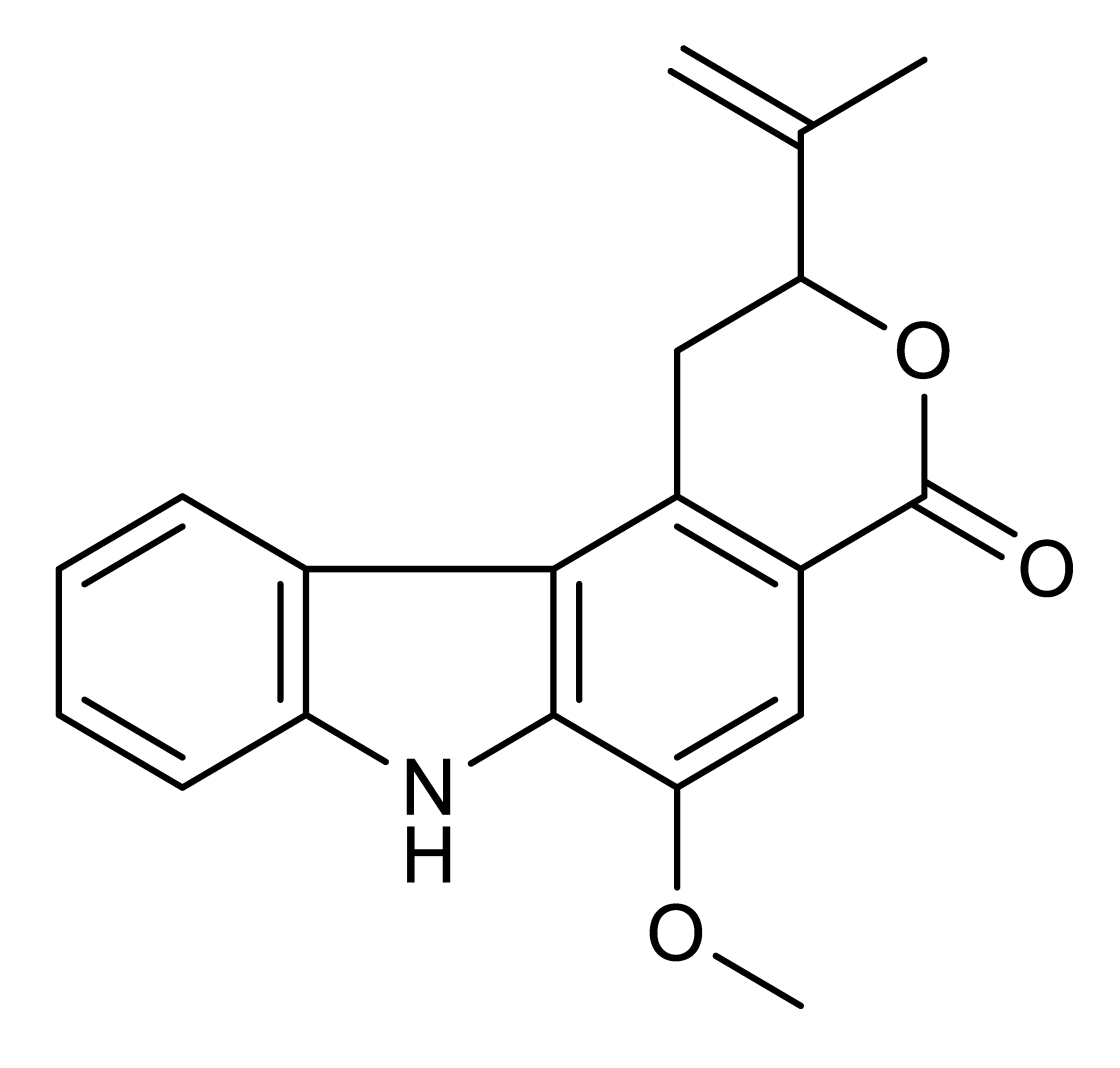	MRSA SK1	Growth inhibition	Species: *Clausena harmandiana*	[69]
Clausine F	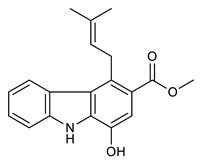	MRSA SK1	Growth inhibition	Species: *Clausena harmandiana*	[69]
2,7-dihydroxy-3-formyl- 1-(3’-methyl-2’-butenyl) carbazole	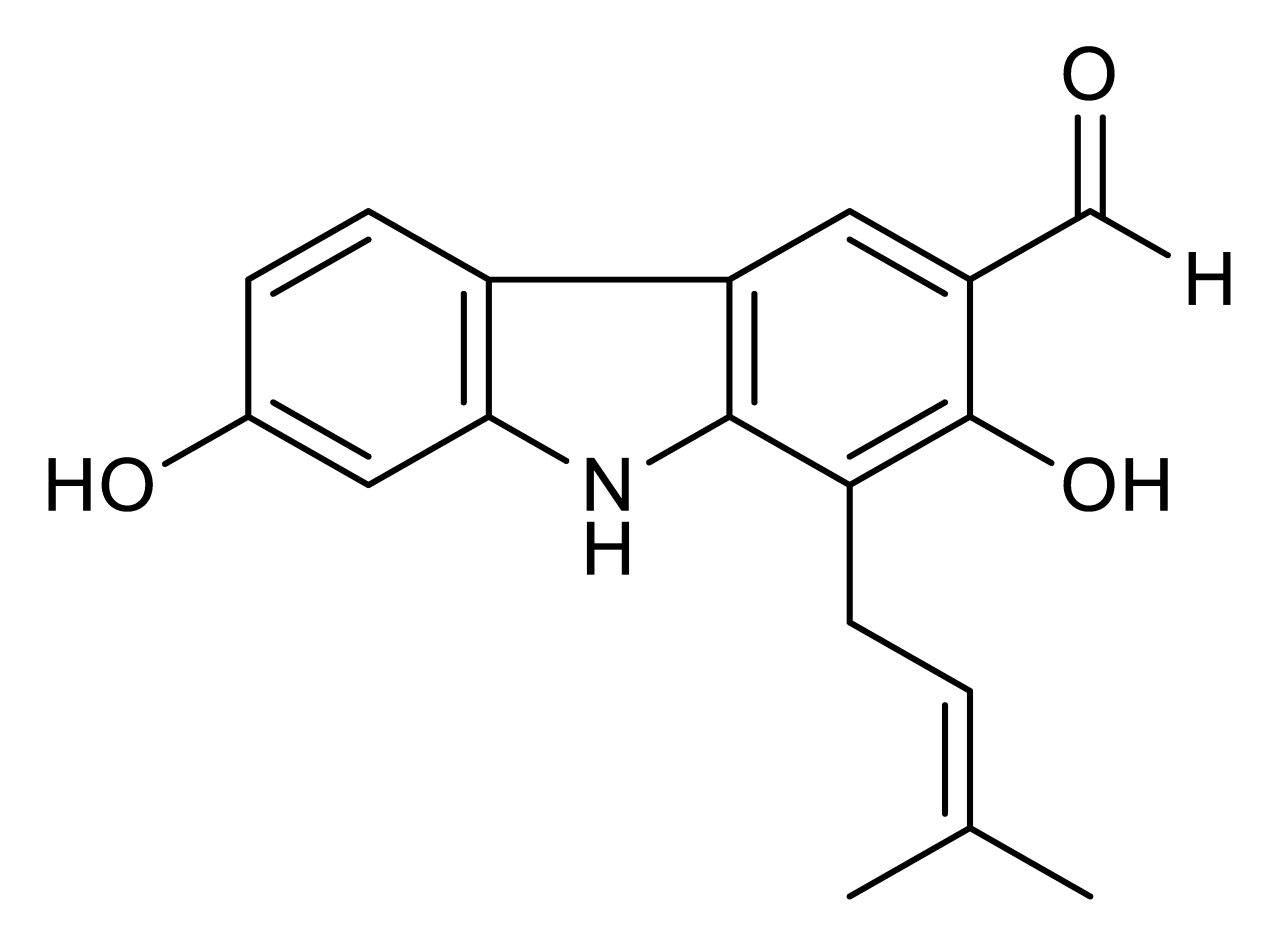	MRSA SK1	Growth inhibition	Species: *Clausena wallichii*	[70]
Clausenawalline E	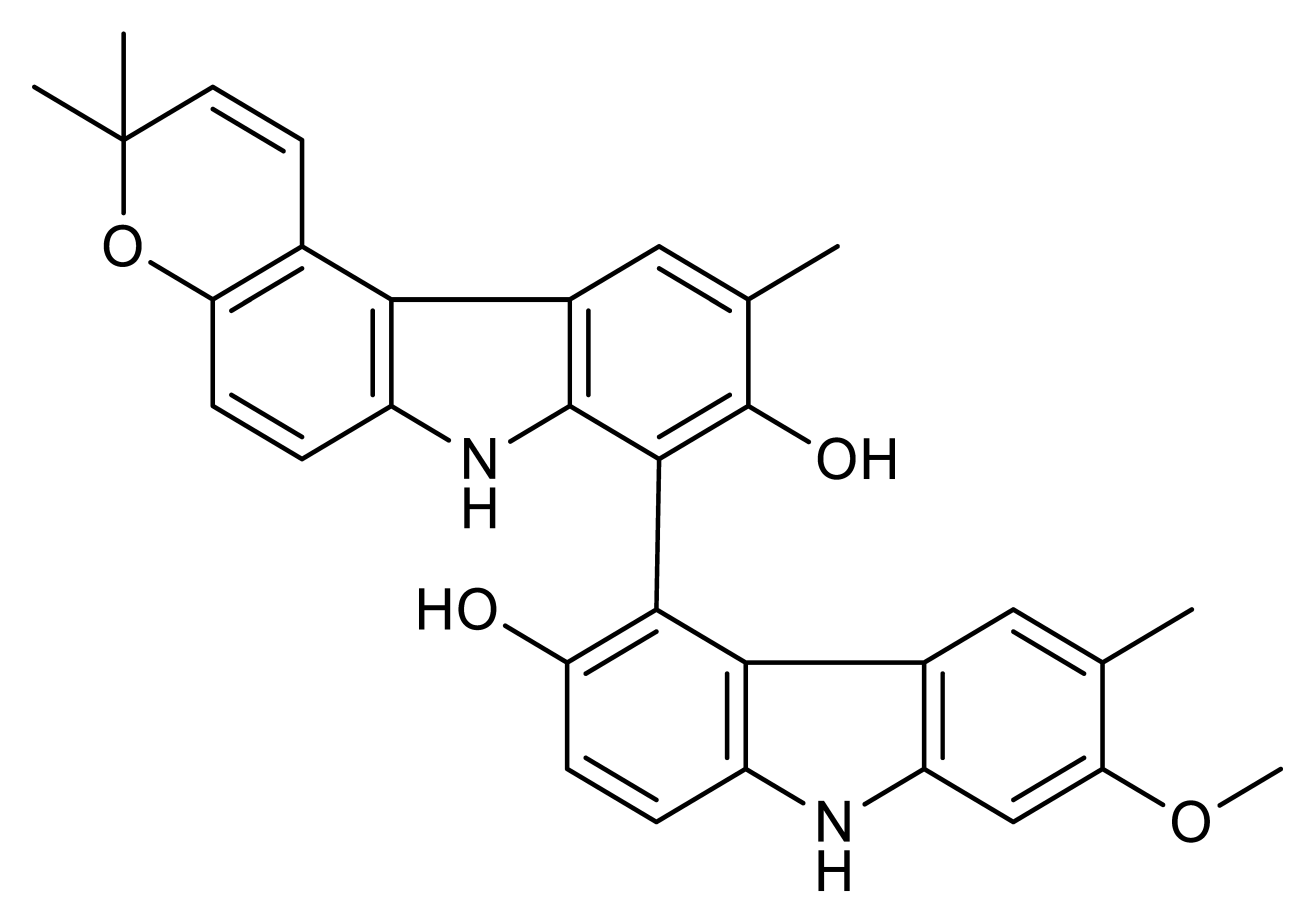	MRSA SK1 *S. aureus*	Growth inhibition	Species: *Clausena wallichii*	[70]
Clausenawalline G	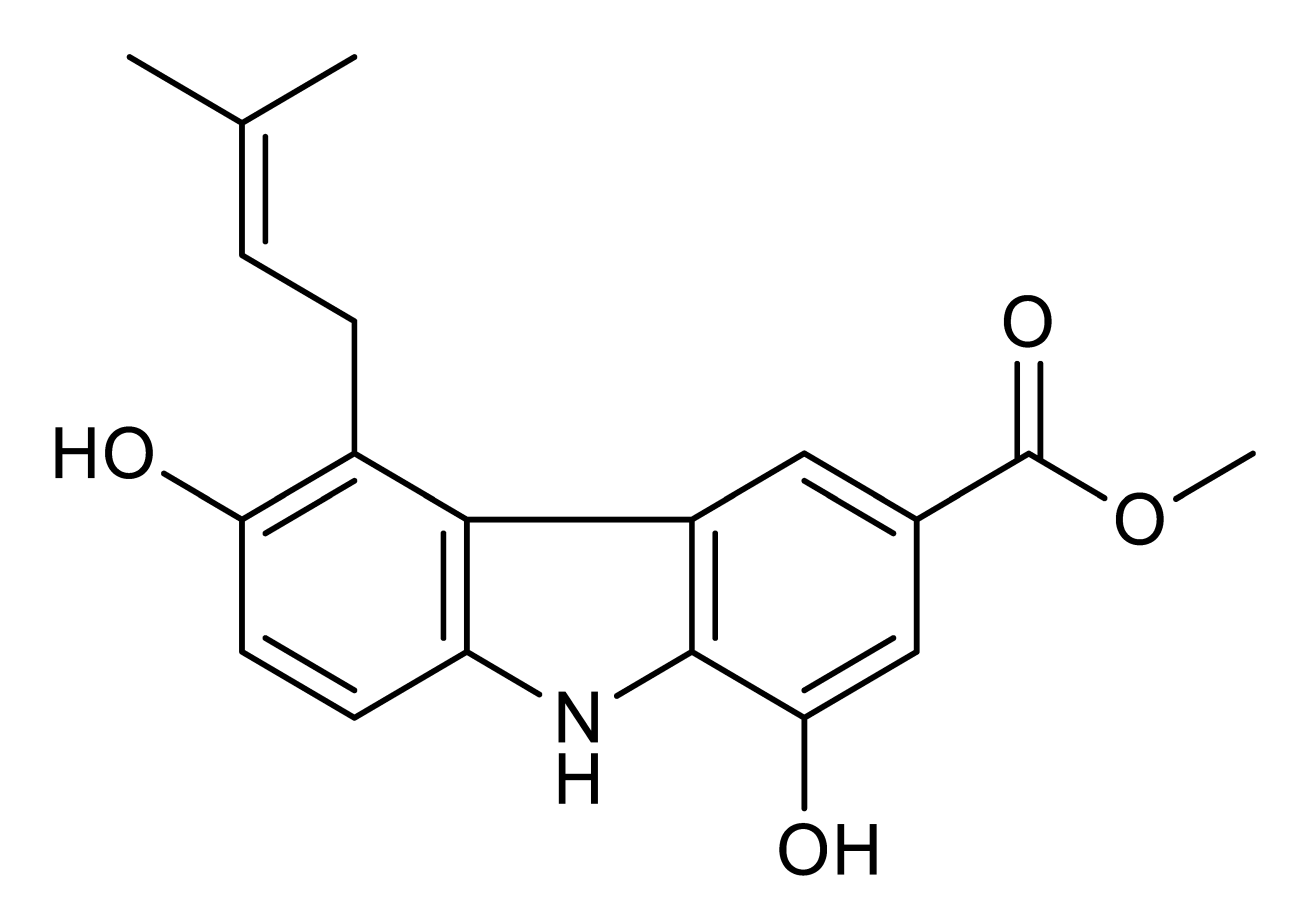	MRSA SK1	Growth inhibition	Species: *Clausena wallichii*	[71]
Clausenawalline H	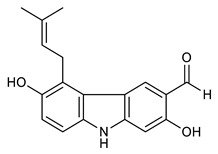	MRSA SK1	Growth inhibition	Species: *Clausena wallichii*	[71]
Clausenawalline I	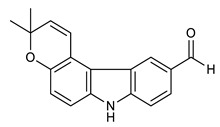	MRSA SK1	Growth inhibition	Species: *Clausena wallichii*	[71]
Clausenawalline J	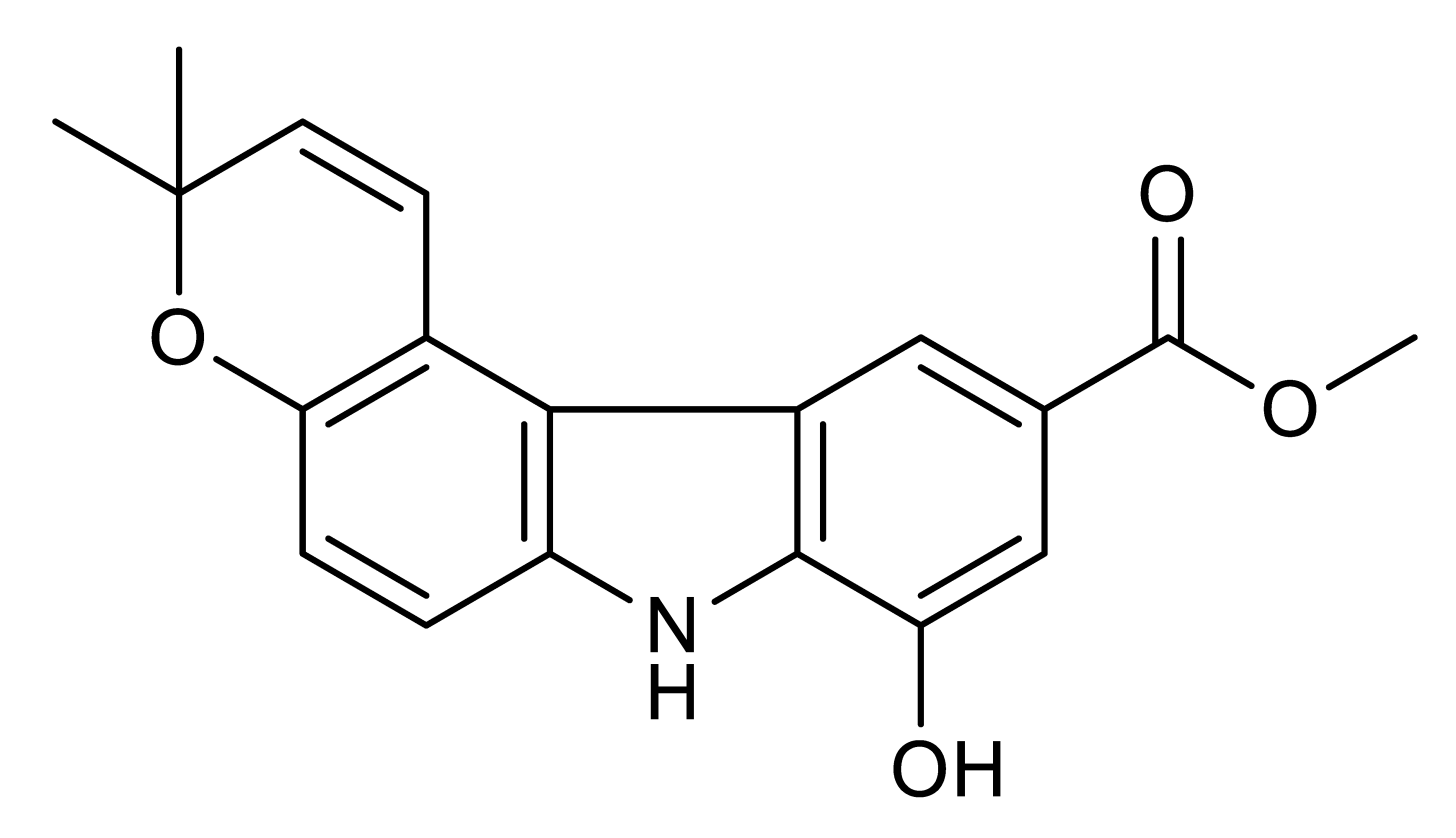	MRSA SK1	Growth inhibition	Species: *Clausena wallichii*	[71]
Clausenawalline K	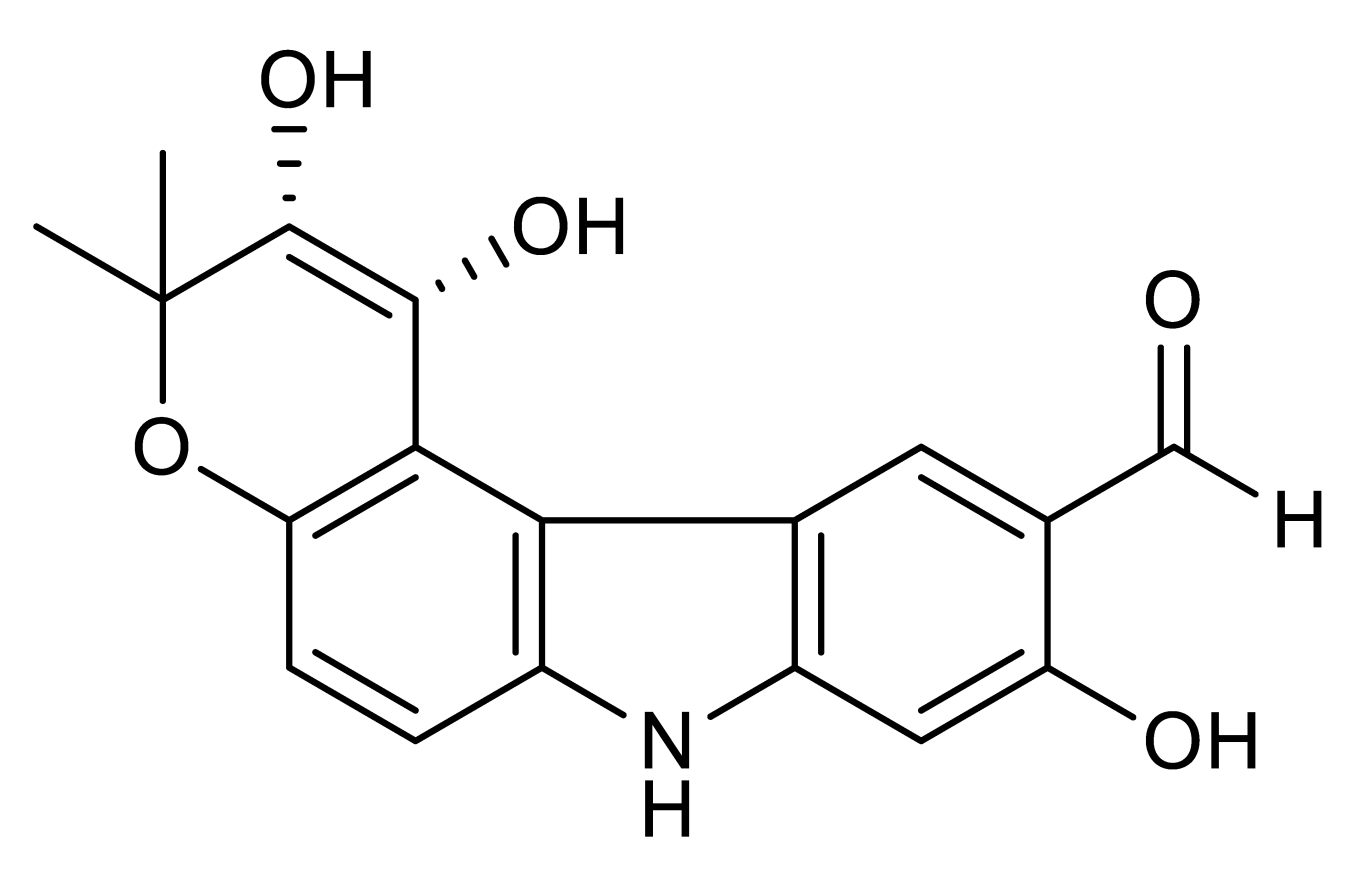	MRSA SK1	Growth inhibition	Species: *Clausena wallichii*	[71]
Mahanine	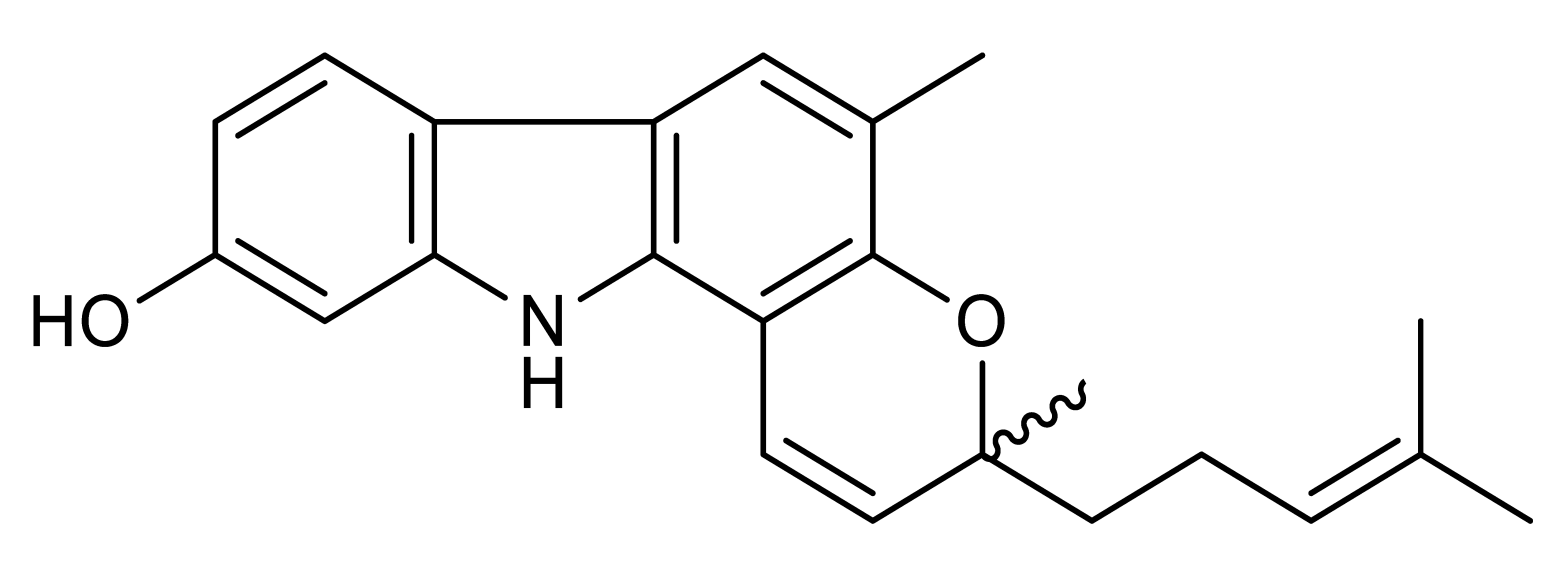	*S. pneumoniae*	Growth inhibition	Species: *Murraya koenigii*	[72]
**Yohimbans**					
Reserpine	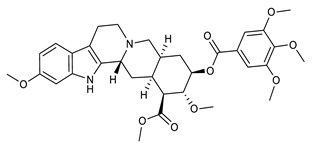	*E* *. coli*	Efflux Pump Inhibitor	Species: *Rauwolfia serpentine*	[73]
**Clavines**					
Lysergol	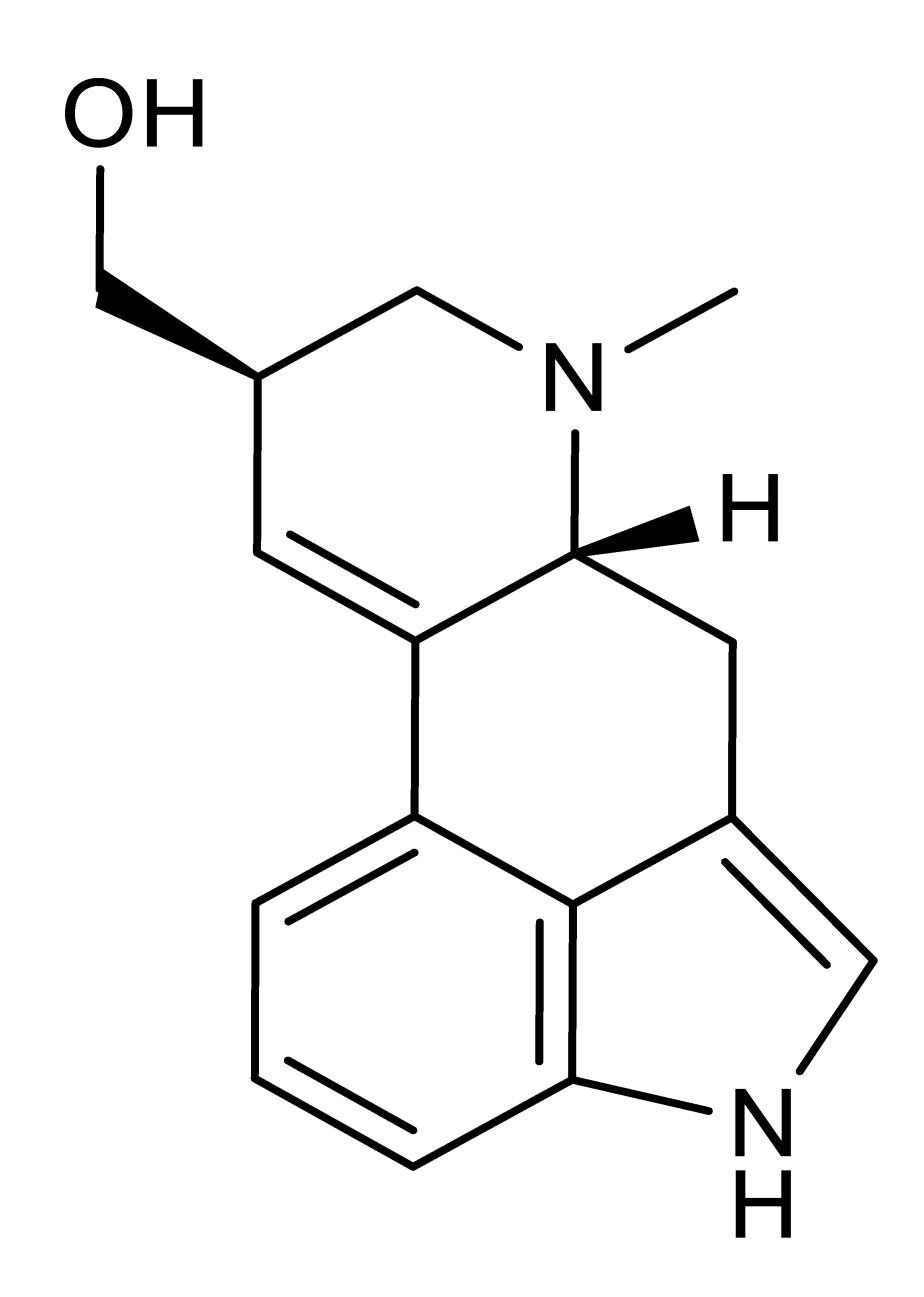	*E. coli*	Efflux Pump Inhibitor	Species: *Ipomoea muricata*	[74,75]
Chanoclavine	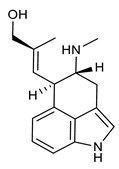	*E. coli*	Efflux Pump Inhibitor	Species: *Ipomoea muricata*	[74,75]
17-O-3″,4″,5″-trimethoxybenzoyllysergol	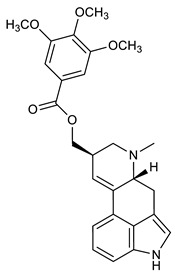	*E. coli*	Efflux Pump Inhibitor	aryl semi-synthetic derivatives	[74]
17-O-3″-nitrobenzoyllysergol	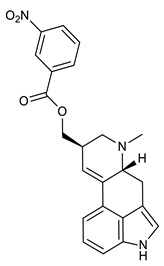	*E. coli*	Efflux Pump Inhibitor	aryl semi-synthetic derivatives	[74]
Indirubin	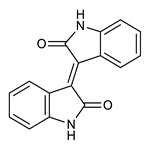	*S. aureus*	Efflux Pump Inhibitor	Species: *Wrightia tinctorial*	[76]

**Table 4:** Summary of antimicrobial activity of some classes of isoquinoline.

Common Name	Chemical Structure	Tested Microorganism	Antimicrobial Effect	Source	Ref.
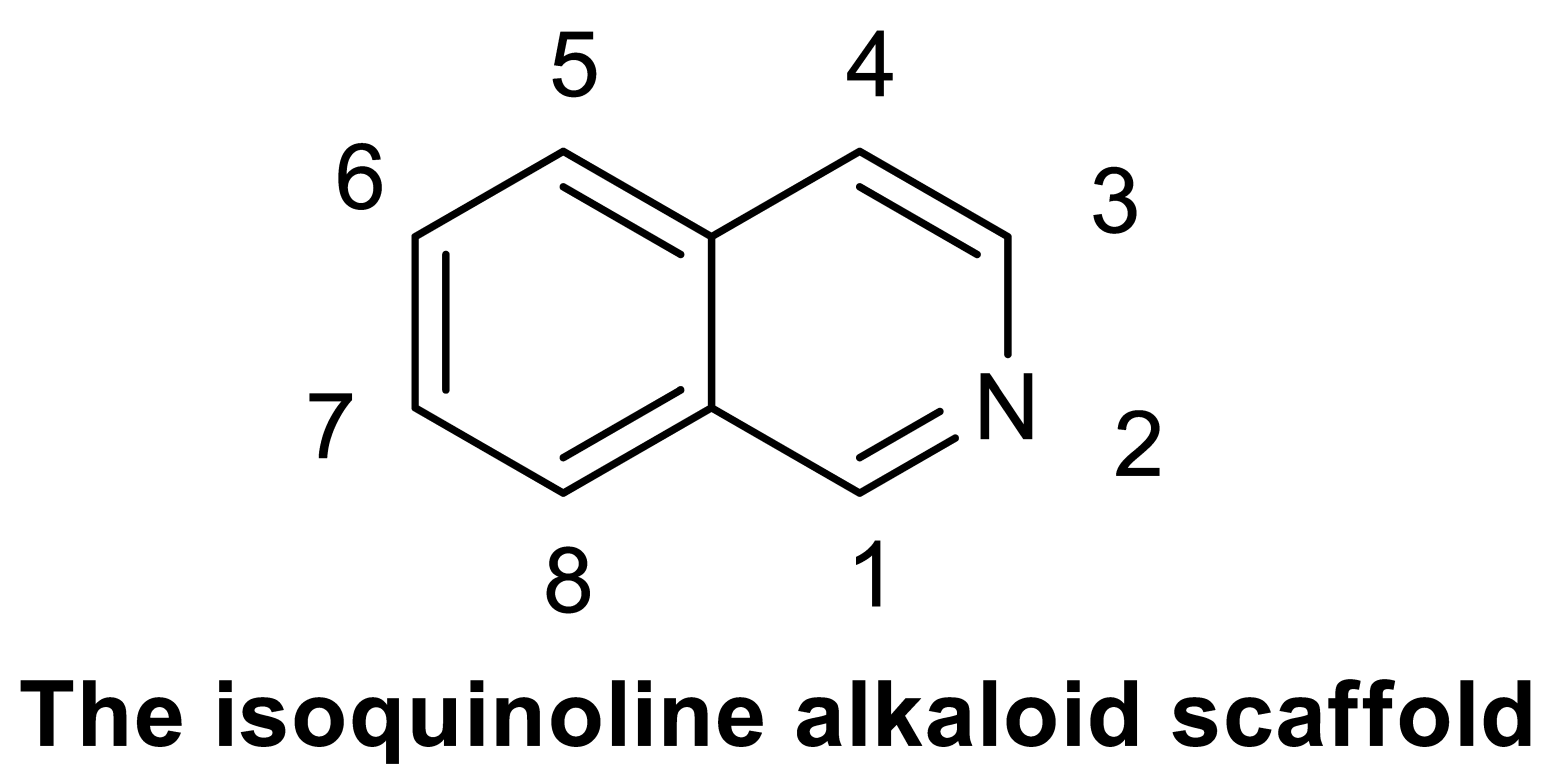					
**Protoberberines**					
Berberine	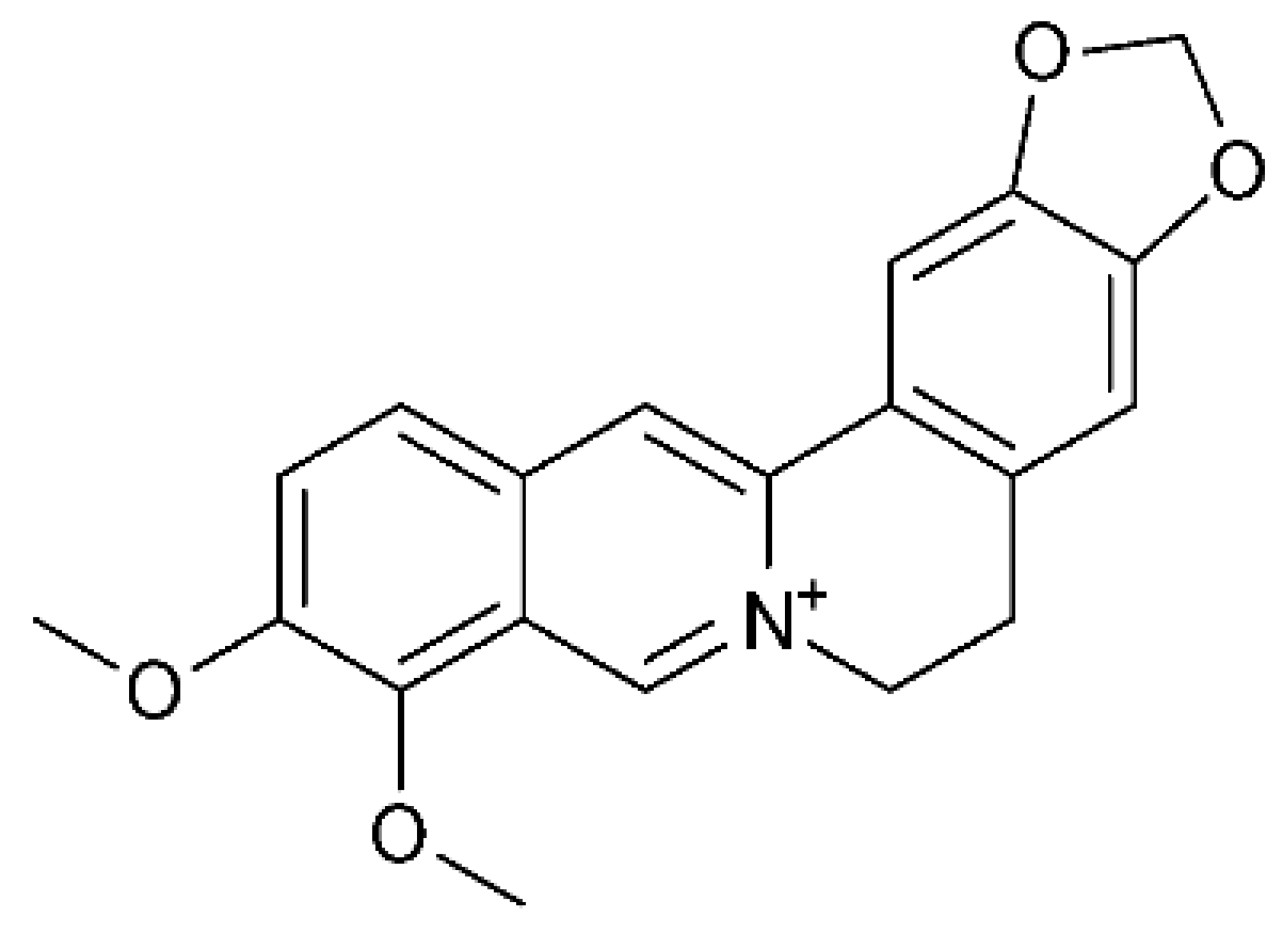	MRSA MSSA *P. aeruginosa*	Efflux Pump Inhibitor, DNA-intercalating	Species: *Berberis* spp.	[100,101]
**Benzophenanthredines**					
Sanguinarine	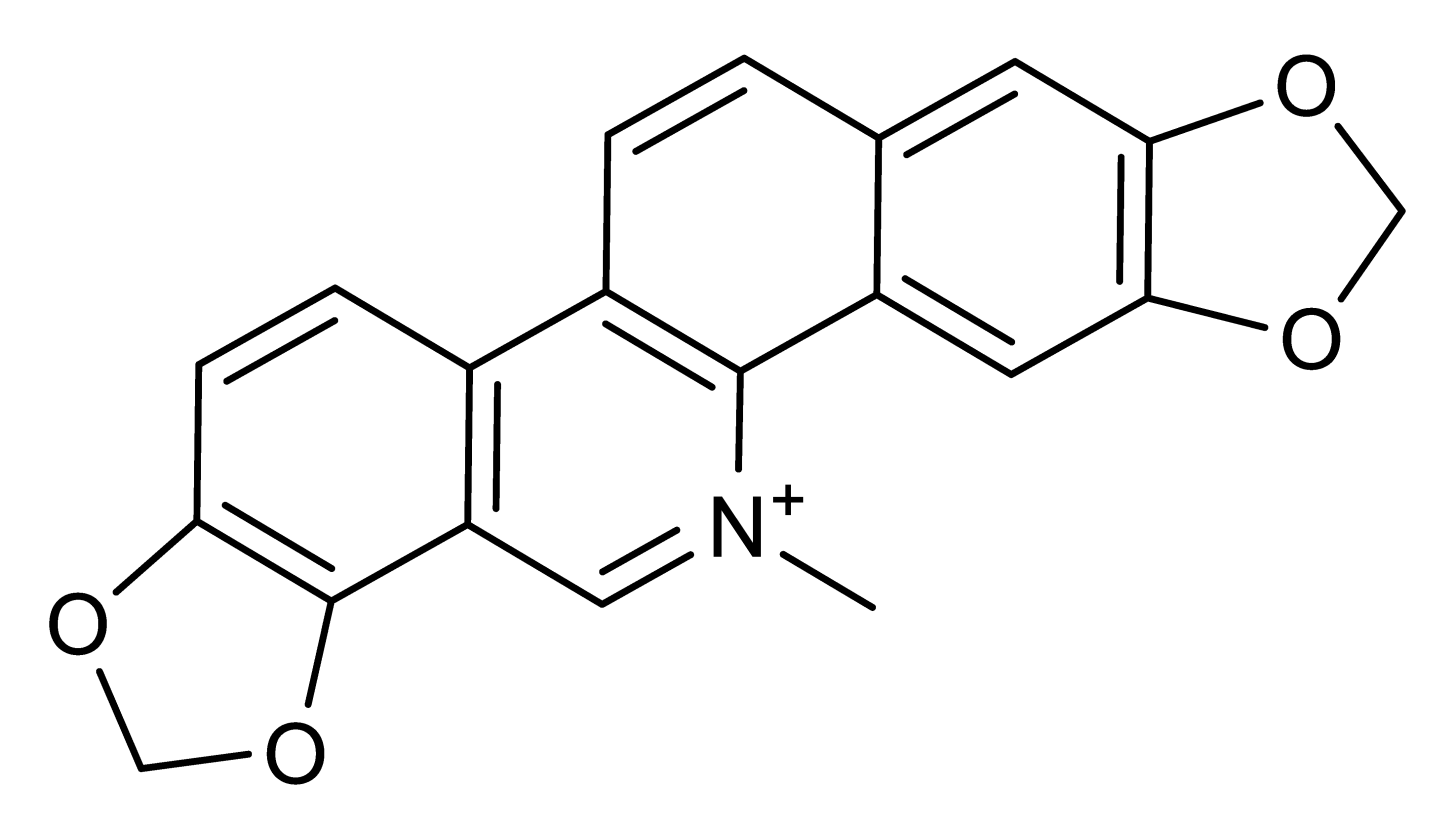	VRE *S. epidermidis*	DNA-intercalating	Species: *Sanguinaria* *canadensis*	[102]
6-Methoxy-dihydrosanguinarine	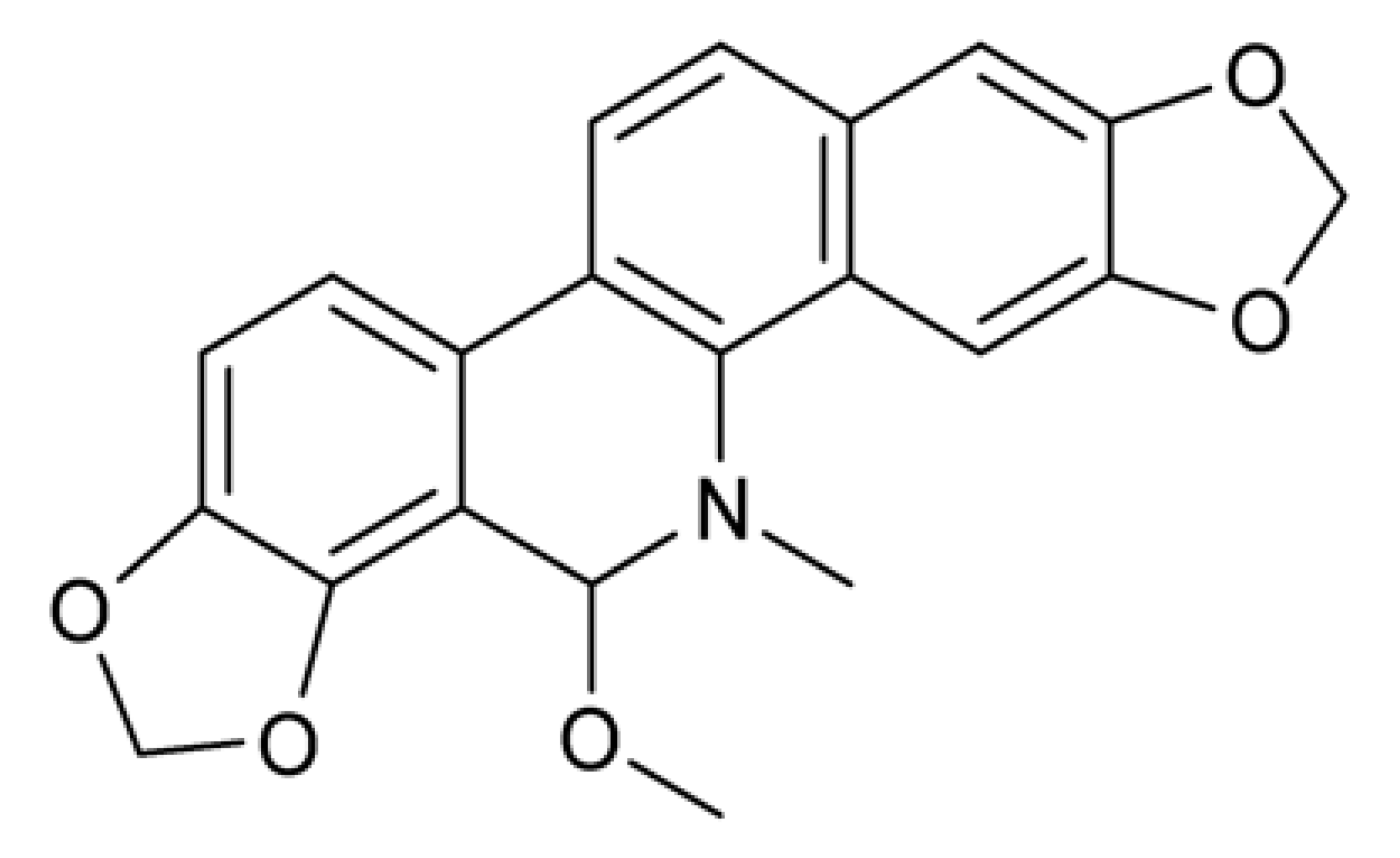	MRSA MSSA	Growth inhibition	Species: *Hylomecon hylomeconoides*	[103]
Chelerythrine	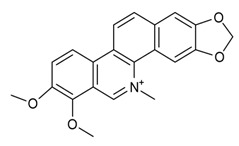	MRSA *S. aureus* ESBLs-SA	Protein biosynthesis inhibitor	Species: *Toddalia asiatica*	[104]
Bis-[6-(5,6-dihydro-chelerythrinyl)] ether	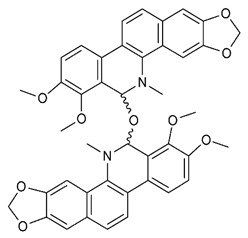	MRSA	Growth inhibition	Species: *Zanthoxylum monophylum*	[105]
6-ethoxy-chelerythrine	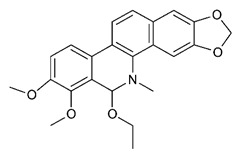	MRSA	Growth inhibition	Species: *Zanthoxylum monophylum*	[105]
Dihydrochelerythrine	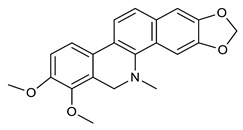	*S. aureus*	Growth inhibition	Species: *Zanthoxylum tingoassuiba*	[106]
Dihydrosanguinarine	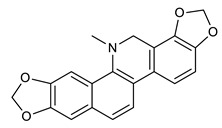	*S. aureus*	Growth inhibition	Species: *Zanthoxylum tingoassuiba*	[106]
N-methylcanadine	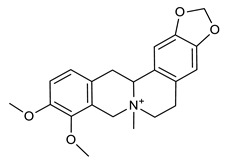	*S. aureus*	Growth inhibition	Species: *Zanthoxylum tingoassuiba*	[106]
6-Hydroxy-dihydrosanguinarine	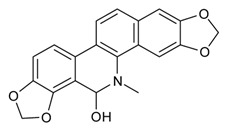	MRSA	Growth inhibition	Species: *Chelidonium maju*	[107]
6-Hydroxy-dihydrochelerythrine	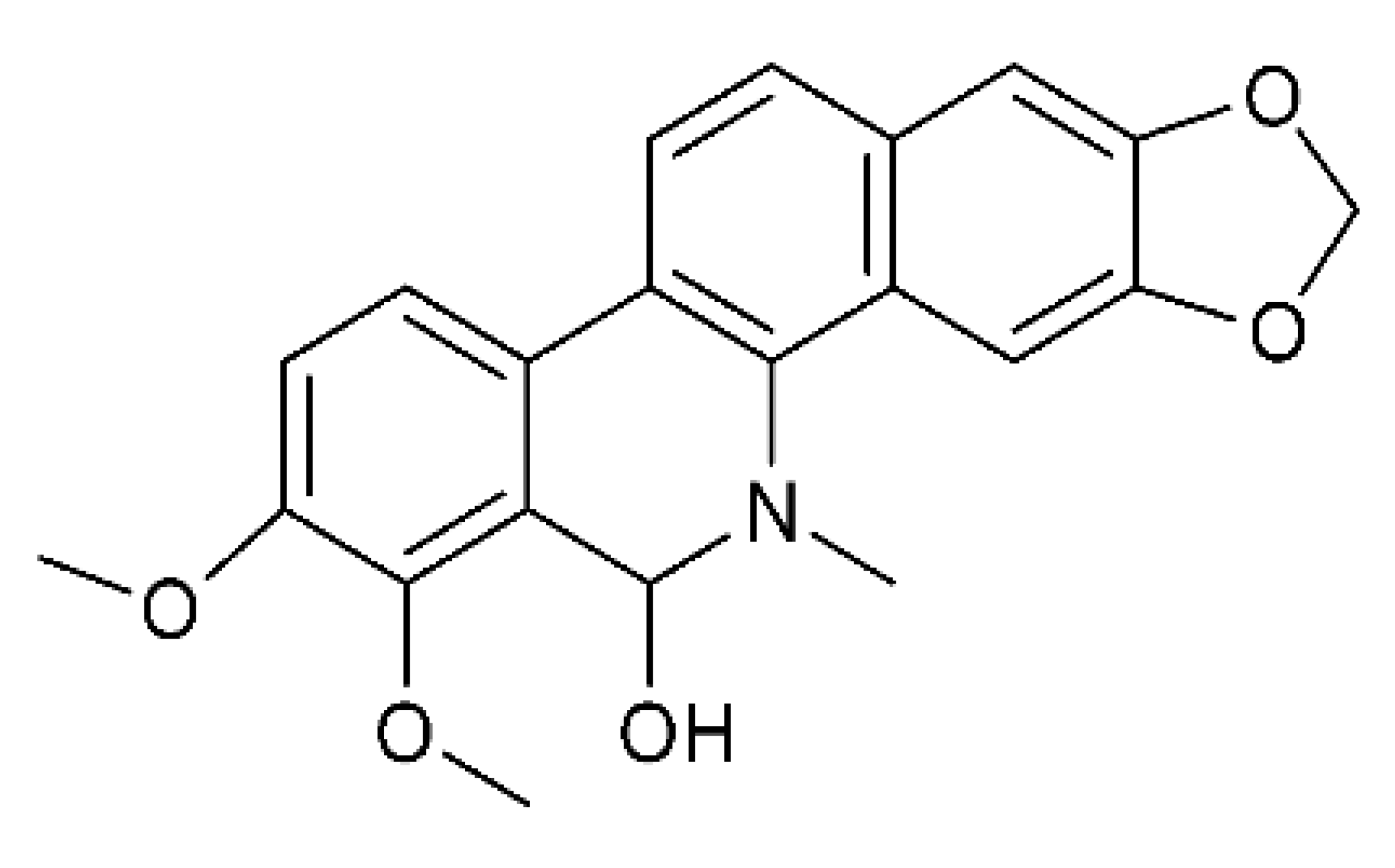	MRSA	Growth inhibition	Species: *Chelidonium maju*	[107]
**Bisbenzylisoquinolines**					
Tetrandrine	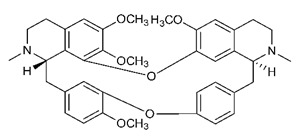	MRSA, ESBL-producing *E. coli*	Growth inhibition	Species: *Stephania tetrandra*	[108,109]
Fangchinoline	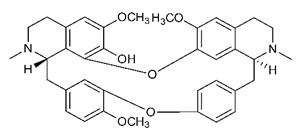	MRSA, ESBL-producing *E. coli*	Growth inhibition	Species: *Stephania tetrandra*	[108,109]
**Aporphines**					
Roemerine	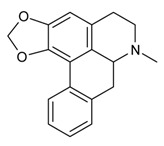	*S. aureus* *B. subtilis*	Efflux Pump Inhibitor Membrane permeability enhancer	Species: *Annona senegalensi, Turkish Papaver* and *Rollinialeptopetal*	[110,111]
